# IFN-**γ**–driven skewing towards Th1 over Th17 differentiation underlies CRS and neutropenia in CAR-T therapy

**DOI:** 10.1172/JCI194631

**Published:** 2025-10-30

**Authors:** Payal Goala, Yongliang Zhang, Nolan Beatty, Allan Pavy, Shannon McSain, Cooper Sailer, Muhammad Junaid Tariq, Showkat Hamid, Eduardo Cortes Gomez, Jianmin Wang, Duna Massillon, Maxwell Ilecki, Justin C. Boucher, Constanza Savid-Frontera, Sae Bom Lee, Hiroshi Kotani, Meredith L. Stone, Michael D. Jain, Marco L. Davila

**Affiliations:** 1 Department of Immunology, Roswell Park Comprehensive Cancer Center, Buffalo, New York, USA.; 2 Cancer Biology PhD Program, University of South Florida, Tampa, Florida, USA.; 3 Iovance Biotherapeutics, San Carlos, California, USA.; 4 Department of Medicine, Roswell Park Comprehensive Cancer Center, Buffalo, New York, USA.; 5 University of Florida College of Medicine, Division of Hematology-Oncology, Gainesville, Florida, USA.; 6 Department of Blood and Marrow Transplant and Cellular Immunotherapy, H. Lee Moffitt Cancer Center & Research Institute, Tampa, Florida, USA.; 7 Department of Biostatistics and Bioinformatics, Roswell Park Comprehensive Cancer Center, Buffalo, New York, USA.; 8 Department of Immunology, H. Lee Moffitt Cancer Center & Research Institute, Tampa, Florida, USA.; 9 Division of Innovative Cancer Control Research, Cancer Research Institute of Kanazawa University, Kanazawa, Japan.

**Keywords:** Hematology, Immunology, Oncology, Cancer immunotherapy, Cytokines, Neutrophils

## Abstract

Chimeric antigen receptor T cell (CAR-T) therapy has led to significant improvements in patient survival. However, a subset of patients experience high-grade toxicities, including cytokine release syndrome (CRS) and immune cell–associated hematological toxicity (ICAHT). We utilized IL-2Ra knockout mice to model toxicities with elevated levels of IL-6, IFN-γ, and TNF-α and increased M1-like macrophages. Onset of CRS was accompanied by a reduction in peripheral blood neutrophils due to disruption of bone marrow neutrophil homeostasis characterized by an increase in apoptotic neutrophils and a decrease in proliferative and mature neutrophils. Both nontumor-bearing and Em-ALL tumor-bearing mice recapitulated the cooccurrence of CRS and neutropenia. IFN-γ–blockade alleviated CRS and neutropenia without affecting CAR-T efficacy. Mechanistically, a Th1-Th17 imbalance was observed to drive cooccurrence of CRS and neutropenia in an IFN-γ–dependent manner leading to decreased IL-17A and G-CSF, neutrophil production, and neutrophil survival. In patients, we observed an increase in the IFN-γ–to–IL-17A ratio in the peripheral blood during high-grade CRS and neutropenia. We have uncovered a biological basis for ICAHT and provide support for the use of IFN-γ blockade to reduce both CRS and neutropenia.

## Abstract 2

Despite clinical success of CAR-T therapy, patients develop toxicities such as cytokine release syndrome and neutropenia, whose co-occurrence impacts their survival and quality-of-life. We recapitulate these toxicities in mice to discover their co-occurrence is driven by Th1-Th17 imbalance following CAR-T administration, which can be prevented via IFNg blockade.

## Graphical abstract

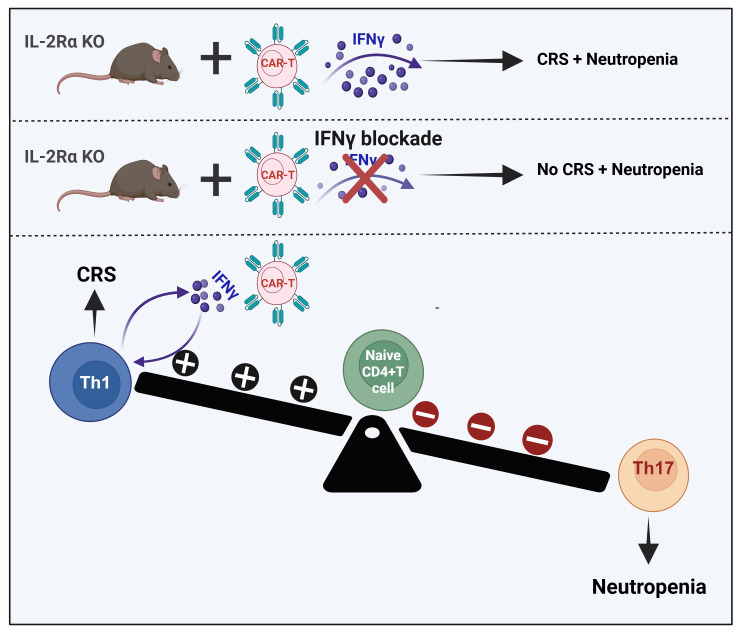

## Introduction

Chimeric antigen receptor T cell (CAR-T) therapy has improved overall and complete response rates, and prolonged progression free survival (PFS) of patients with relapsed/refractory hematological malignancies (1). The short-term and long-term impacts of anti-CD19 CAR-T products on the immune landscape of B cell malignancies has garnered considerable attention in recent years (2, 3), most notably pertaining to CAR-T associated toxicities, which include cytokine release syndrome (CRS) and immune cell associated neurotoxicity syndrome (ICANS). CRS arises from the excessive release of cytokines such as IFN-γ and TNF-α (4) from CAR-T cells that activate macrophages and other immune cells to release more proinflammatory cytokines, which trigger a cascade of events leading to adverse events such as fever, hypotension, hypoxia, and organ toxicities (2). Overactivation of ANG-2 and Von Willebrand factor during this process causes endothelial dysfunction that is associated with ICANS (5). Recent clinical studies have shown that CAR-T–treated patients also suffer from cytopenias (6). The mechanisms leading to CAR-T–associated cytopenia remains elusive. Rejeski et al. showed that severe cytopenia in patients after CAR-T treatment led to frequent infections and increased hospitalizations (7, 8). Onset of cytopenia was associated with worse survival of patients after CAR-T treatment, thereby emphasizing the need to address these hematological toxicities (6). An observation drawn from 2 independent clinical trials was the cooccurrence of cytopenia with CRS (6, 9). Among the cytopenias, neutropenia was most predominant (6), where the decline in neutrophils intensified with the grade of CRS (7, 9). These prolonged cytopenias have been labelled as immune cell–associated hematologic toxicity (ICAHT) and are a substantial cause of nonrelapse mortality (NRM), but with a lack of effective treatments and little known about their underlying cause (10–12).

High disease burden and excess CAR-T expansion are major factors leading to inflammation that drives CAR-T toxicities (2, 13). Fischer et al. showed that patients with multiple myeloma (MM) with a smaller proportion (among T cells) of CD25^+^CD127^low^ Regulatory T cells (Tregs) at lymphodepletion were associated with improved response to CAR-T treatment and also had a higher maximum C-reactive protein (CRP; an indicator of CRS) (14). Firestone et al. noted that patients with MM who experienced CRS following treatment with a bispecific T cell engager had significantly longer PFS, where a smaller pretreatment Treg (CD25^+^CD127^low^) proportion among T cells (and an increase in CD8^+^ T cell proportion) was found to be one of the major drivers for improved response (15). Kitamura et al. confirmed a direct correlation between onset of CRS and a significant decrease in Tregs (CD4^+^CD25^+^CD127^-/low^ T cells) (16). These clinical findings suggest an association between CRS, response to T cell therapies, and Tregs (CD25^+^CD127^low^). IL-2Ra (also known as CD25), a key mediator of lymphoproliferation, is essential for Treg expansion and survival (17, 18) so we used in vivo IL-2Ra knockout (KO) mice to model toxicities during CAR-T treatment.

## Results

### IL-2Ra KO mice recapitulate CRS following CAR-T administration.

**Figure 1:**
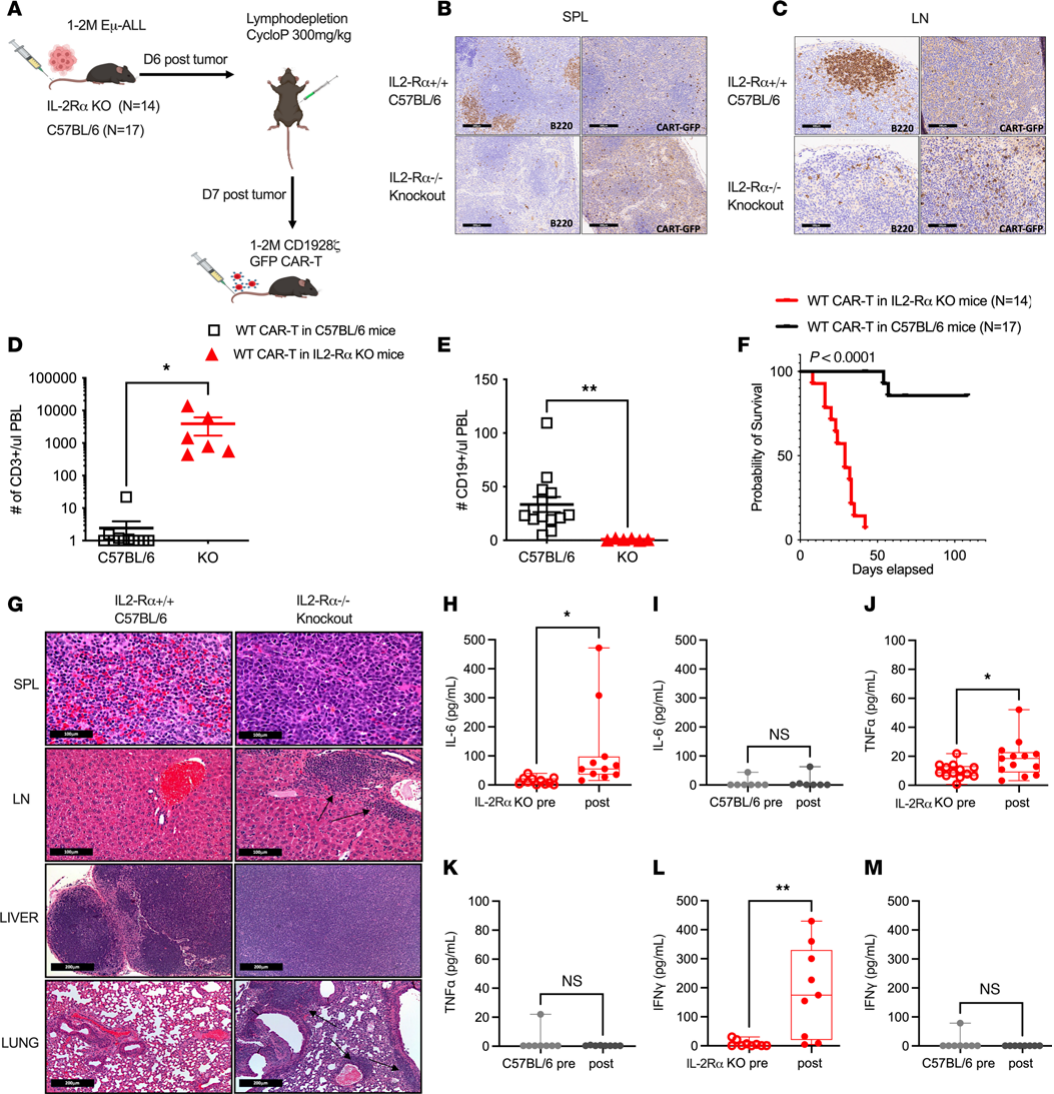
IL-2Ra KO mice recapitulate CRS following CAR-T administration. (**A**) Schematic representation of IL-2Ra knockout (KO) or C57BL/6J mice inoculated with 1 to 2 Million (M) Em-ALL cells followed by 300 mg/kg cyclophosphamide (CycloP) and 1 to 2 M CD1928z GFP WT CAR-T infusion. Data is pooled from 2 independently performed experiments. (**B**) Histopathology sections of Spleen (SPL) at 100× (black scale bar: 200 mm) and (**C**) Lymph node (LN) at 400× (black scale bar: 100 mm) a week after WT CAR-T infiltration in IL-2Ra KO vs control (C57BL/6J) mice. The panel to the left shows B220+ cells (brown) and panel to the right shows WT CAR-T cells (brown). (**D**) The percentage of CD3+ and (**E**) the percentage of CD19+ cells in peripheral blood (PBL) were compared between a subset of IL-2Ra KO (*n* = 6) and C57BL/6J (*n* = 14) mice at week 1 after WT CAR-T administration. (**F**) Kaplan-Meier overall survival curves of IL-2Ra KO vs C57BL/6J mice treated with CD1928z WT CAR-T cells. (**G**) H&E-stained sections of (Top to Bottom) SPL, LN (at 400×; black scale bar: 100 mm), Liver and lung (at 100×; black scale bar: 200 mm) from IL-2Ra KO and C57BL/6J mice at week 1 after WT CAR-T infusion. Arrows shown in LN and lung sections in **E** represent a mixed leukocyte infiltration including large histiocytic cells. Week 1 serum cytokine analysis of IL-6 (**H** and **I**), TNF-α (**J** and **K**) and IFN-γ (**L** and **M**) in WT CAR-T–treated IL-2Ra KO and C57BL/6J mice, respectively. In **I**, **K**, and **M**, cytokines were measured for 7 C57BL/6J mice, while in (**H**, **J**, and **L**), cytokines were measured for all IL-2Ra KO mice that had samples for both pre and postcytokines, as paired values from each mouse. Error bars represent standard error of mean (SEM). *P < 0.05, **P < 0.01, and ***P < 0.001. *P* values for cytokine bar plots (**D**, **E**, and **H**–**M**) were generated using a paired *t* test. *P* values for Kaplan-Meier survival curve **F** was generated using Log-rank (Mantel-Cox) test.

Two important factors affecting the severity of CRS in patients after CAR-T therapy are the extent of CAR-T proliferation and the release of proinflammatory cytokines (3). We hypothesized that knockout of IL-2Ra can model the proinflammatory environment during CRS due to the relationship between Treg depletion and systemic release of proinflammatory cytokines, which was shown by Gogishvili et al. (19). We inoculated IL-2Ra knockout (KO) mice as well as C57BL/6J control mice with the syngeneic malignant B cell line, Em-ALL (20). A week after tumor engraftment, IL-2Ra KO and C57BL/6J mice were administered cyclophosphamide and a day later CD1928z CAR-T cells that were derived using WT Thy 1.1 mice (that express IL-2Ra) (Figure 1A). WT CAR-T expansion was confirmed in IL-2Ra KO mice by the presence of GFP+ CAR-T cells in spleen and lymph node. The extent of WT CAR-T infiltration was significantly higher in IL-2Ra KO mice compared with WT and this correlated with greater B cell (B220+) aplasia (Figure 1, B and C). Enhanced WT CAR-T expansion was also observed in peripheral blood of IL-2Ra KO compared with WT mice, which led to improved efficacy in B cell killing (Figure 1, D and E). IL-2Ra KO mice treated with WT CAR-T displayed poor survival of less than 35 days (*P* < 0.0001) compared with C57BL/6J mice that survived more than 100 days (Figure 1F). Laboratory analysis demonstrated that these deaths were due to CRS and not progressive cancer. We identified infiltration of leukocytes and histiocytes in the H & E–stained sections of splenic red pulp, lymph node, liver, and lung tissues (Figure 1G). Evaluation of serum cytokines at week 1 after WT CAR-T administration in IL-2Ra KO mice confirmed the significant elevation of proinflammatory cytokines IL-6, TNF-α and IFN-γ (Figure 1, H, J, and L). There were no notable differences in serum cytokines of WT CAR-T treated C57BL/6J WT mice (Figure 1, I, K, and M).

### Reversal of CRS phenotype in IL-2Ra KO mice via IL-6R-blockade, IFN-γ blockade, and Treg adoptive transfer.

**Figure 2:**
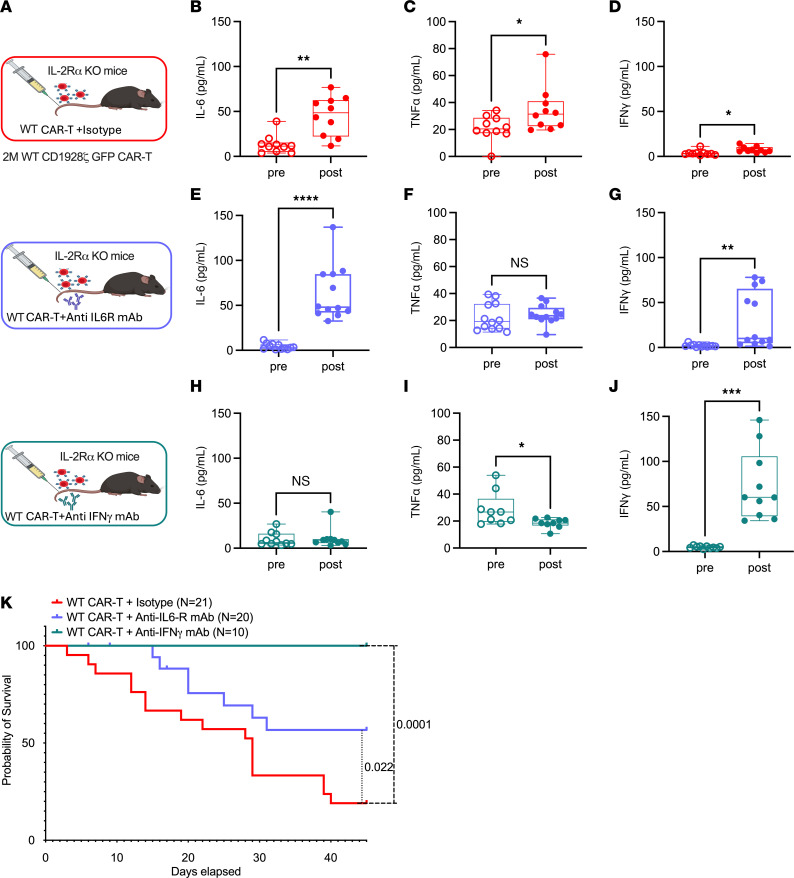
Mitigating CRS in IL-2Ra KO mice. (**A**) Schematic representation of 51 IL-2Ra KO mice treated with 2M WT CAR-T followed by administration of either Isotype IgG or 12.5 mg/kg (i.p.) of anti-IL-6R mAb or anti-IFN-γ mAb on a twice weekly basis. Data is pooled from 2 independently performed experiments. (**B**–**J**) Serum analysis in pre versus week 4 after WT CAR-T treated mice of cytokines IL-6 (**B**, **E**, and **H**), TNF-α (**C**, **F**, and **I**) and IFN-γ (**D**, **G**, and **J**). Pre and postcytokines are paired values taken from each mouse alive at week 4 for each cohort (*n* = 10 for WT CAR-T + Isotype, *n* = 12 for CAR-T + anti-IL-6R mAb and *n* = 10 for WT CAR-T + anti-IFN-γ mAb). (**K**) The impact of CRS management in IL-2Ra KO mice was determined by measuring Kaplan-Meier overall survival. Error bars represent SEM. *P < 0.05, **P < 0.01, and ***P < 0.001. *P* values for cytokine bar plots (**B**–**J**) were generated using paired *t* test. *P* values for Kaplan-Meier survival curve (**K**) was generated using Log-rank (Mantel-Cox) test.

While lymphodepletion followed by WT CAR-T treatment best reflects clinical practice, it also confounds the role of CAR-T on the endogenous immune landscape. Therefore, we treated IL-2Ra KO and C57BL/6J mice with WT CAR-T cells only, i.e., without any tumors or lymphodepleting agent cyclophosphamide (Figure 2A). A significant elevation of CRS associated proinflammatory cytokines IL-6 (*P* = 0.003), TNF-α (*P* = 0.041) and IFN-γ (*P* = 0.027) were observed in IL-2Ra KO mice after 4 weeks of WT CAR-T treatment (Figure 2, B–D). Compared with Figure 1, lack of tumors could influence the levels of tumor-associated inflammatory cytokines such as IFN-γ. To validate CAR-T–associated CRS, IL-2Ra KO mice were treated with either anti–IL-6R monoclonal antibody (mAb) or anti–IFN-γ mAb, which are equivalents for Tocilizumab and Emapalumab, respectively. Tocilizumab is used to manage CRS in a clinical setting, whereas Emapalumab has recently been shown to mitigate CRS also (21). Both anti–IL-6R and anti–IFN-γ mAbs were administered twice every week, followed by weekly collection of peripheral blood for complete blood profiling and serum analysis until endpoint. Compared with WT CAR-T–only treatment, we report baseline restoration of cytokines TNF-α, whereas IL-6 and IFN-γ levels remained high in IL-2Ra KO mice after treatment with anti–IL-6R mAb (Figure 2, E–G). Treatment with anti–IFN-γ mAb (Figure 2, H–J) reduced IL-6 and TNF-α, but we again noted an increase in IFN-γ. We note that anti–IL-6R mAb treatment can be a confounding factor in the measurement of IL-6 (Figure 2E), (22, 23). However, since IL-6 is a key component of CRS assessment, we measured it. Similarly, increase in IFN-γ (Figure 2J) following treatment with anti–IFN-γ mAb can be a consequence of target-mediated drug disposition (TMDD) (24–26). To evaluate this, we measured CXCL9, which is induced by IFN-γ and is an established marker for functional measurement of IFN-γ neutralization (24–26).While WT CAR-T treatment showed a significant increase in CXCL9 (*P* = 0.007), administration of anti–IFN-γ mAb led to a significant inhibition of CXCL9 (*P* < 0.0001), thereby confirming the abatement of IFN-γ signaling (Supplemental Figure 1, A–D; supplemental material available online with this article; https://doi.org/10.1172/JCI194631DS1). A 6-week survival analysis of IL-2Ra KO mice (Figure 2A) treated with WT CAR-T showed a significant decrease in survival compared with anti-IL-6R mAb (*P* = 0.022) and anti–IFN-γ mAb (*P* = 0.0001) groups (Figure 2K).

IL-2Rα KO mice have dysfunctional Tregs and we hypothesized that CRS in IL-2Ra KO mice after CAR-T therapy could in part be due to this deficiency. Clinical studies have similarly shown that reduced Treg frequency accounted for higher rate of CAR-T–associated ICANS (27) and CRS (14, 15). IL-2Ra KO mice were treated with WT CAR-T alone (Group 1), or in combination with adoptively transferred low dose Tregs (0.25 M; Group 2), or high dose Tregs (1 M; Group 3) infused prior to WT CAR-T (Supplemental Figure 2A). Lower Treg dosing was better at improving survival of mice (WT CAR-T+0.25M Tregs versus WT CAR-T alone: *P* = 0.056) compared with higher Tregs (WT CAR-T+1M Tregs versus WT CAR-T alone: *P* = 0.265) (Supplemental Figure 2B). Compared with WT CAR-T alone (Supplemental Figure 2, C–E), low and high dose Tregs reduced IL-6, TNF-α and IFN-γ to baseline (Supplemental Figure 2, F–K). We believe poor survival of mice with high dose Tregs can be due to the reported effect of lymphopenia-induced cell death (28).

### IL-2Ra KO mice show cooccurrence of CRS and neutropenia following CAR-T infusion.

**Figure 3:**
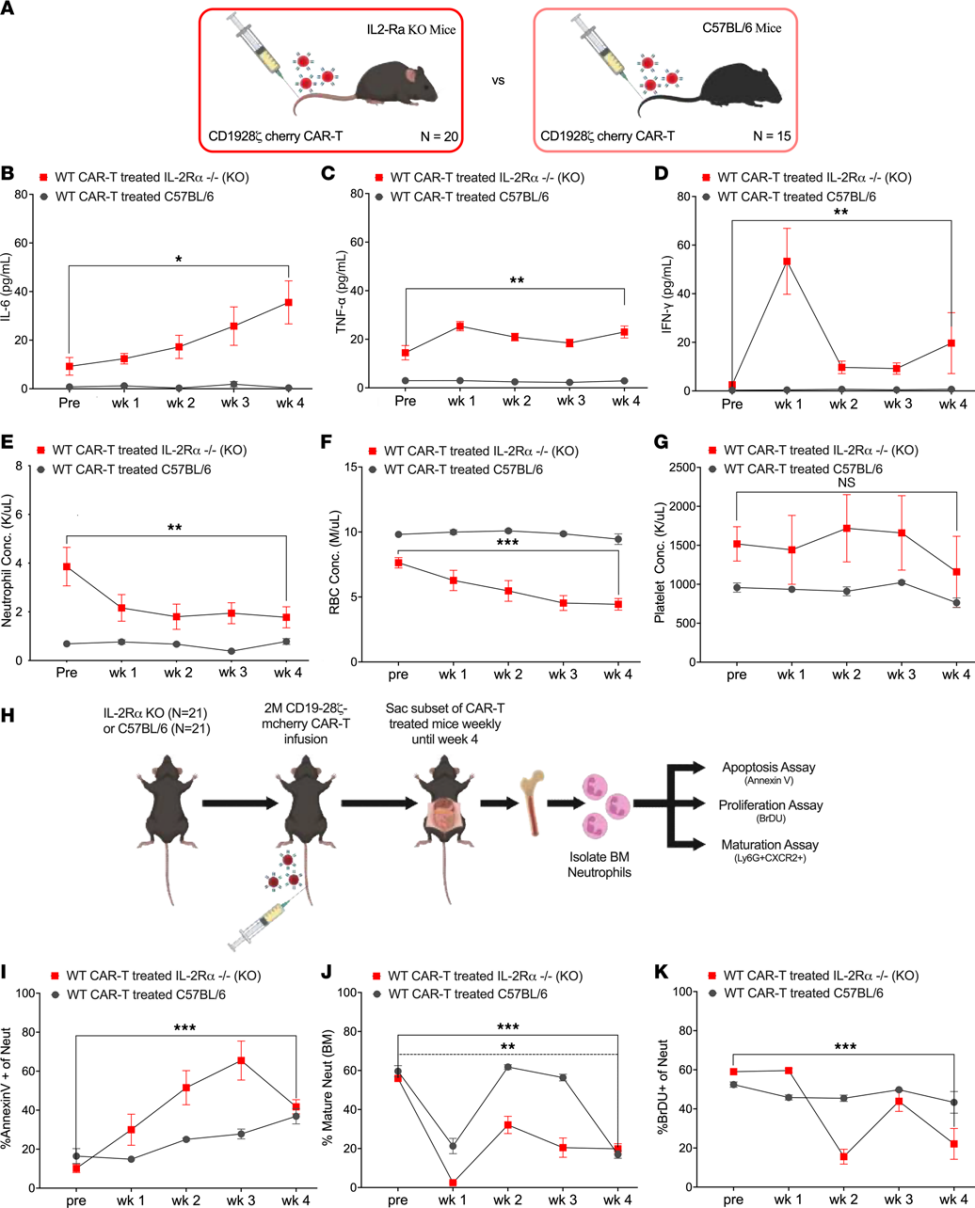
Evaluating cooccurrence of CRS and Neutropenia in IL-2Ra KO mice. (**A**) Schematic representation of IL-2Ra KO and WT mice treated with 2M WT CAR-T (i.v.) cells. Data are pooled from 2 independently performed experiments. (**B**–**D**) Time course analysis of cytokines IL-6 (**B**), TNF-α (**C**) and IFN-γ (**D**) in post WT CAR-T treated IL-2Ra KO and WT mice. IL-2Ra KO pre and postcytokines are paired values taken from each mouse alive until week 4 (*n* = 10). WT paired cytokine values represent a subset of live mice until week 4 (*n* = 7). (**E**–**G**) Time course of neutrophil concentration (K/mL) (**E**), red blood cell (RBC) concentration (M/mL) (**F**) and platelet concentration (K/mL) (**G**) in the peripheral blood from IL-2Ra KO and C57BL/6J mice using complete blood profiling (CBC). Pre and post levels from CBC are paired values taken from each mouse alive until week 4 per cohort (*n* = 8 for IL-2Ra KO and *n* = 8 for C57BL/6J). (**H**) Schematic of IL-2Ra KO and C57BL/6J mice treated with WT CAR-T cells sacrificed periodically followed by kit-based isolation of neutrophils from bone marrow (BM). Cell turnover of the purified neutrophils was analyzed for apoptosis using Annexin V, and proliferation using BrDU. Neutrophil maturation rate was analyzed using BMMC. Data are pooled from 2 independently performed experiments. **I**–**J**. Time point comparison in IL-2Ra KO versus C57BL/6J mice of % Annexin V+ apoptotic cells as a frequency of neutrophils (kit-based purification) (**I**) and % Ly6G+CXCR2+ cells as a frequency of mature neutrophils (gated on BMMC as Live+Lineage-CD11b+Gr1+ckit-CXCR4- cells) (**J**). (**K**) IL-2Ra KO and C57BL/6J mice were injected with 2mg BrDU i.p. 48 hours prior to harvest, followed by staining for BrDU-labelled neutrophils. Line plot represents % BrDU+ cells as a frequency of live neutrophils (kit-based purification) at weeks 1 through 4. Error bars represent SEM. *P < 0.05, **P < 0.01, and ***P < 0.001. *P* values for line plots (**B**–**G**) were generated using paired *t* test. *P* values for line plots (**I**–**K**) were generated using unpaired *t* test.

Higher incidence of cytopenias are reported in patients who develop CRS (6, 9). Longitudinal analysis of IL-2Ra KO mice compared with C57BL/6J mice, after WT CAR-T treatment (Figure 3A), showed elevation of IL-6 (*P* = 0.03), TNF-α (*P* = 0.008) and IFN-γ (*P* = 0.001) (Figure 3, B–D), while complete blood profiling showed significant decline in neutrophils (*P* = 0.007) and red blood cells (Figure 3, E and F). However, a consistent pattern of thrombocytopenia could not be confirmed in IL-2Ra KO mice (Figure 3G). Neither neutropenia, anemia, nor thrombocytopenia were observed in C57BL/6J mice. To determine the extent of CAR-T–associated neutropenia, we compared neutrophil levels of IL-2Ra KO mice treated with WT CAR-T alone, or lymphodepletion alone, or in combination (Supplemental Figure 3A). Both WT CAR-T alone and lymphodepletion alone led to a drop in neutrophils within one week; however, lymphodepletion alone showed a rapid recovery in neutrophils within 5 weeks and WT CAR-T alone had prolonged neutropenia (*P* = 0.004) (Supplemental Figure 3B). Lymphodepletion followed by WT CAR-T similarly showed poor recovery in neutrophils (*P* = 0.047).

Neutrophils are developed in the bone marrow following granulopoiesis (29, 30). Since neutrophils are short lived, a healthy pool of mature (CD11b+Ly6G+CXCR2+) neutrophils in the bone marrow is essential to maintain circulating neutrophils (31). Additionally, apoptotic neutrophils are homed back to the bone marrow for elimination (32). To determine the effect of WT CAR-T cells on neutrophil homeostasis, IL-2Ra KO and C57BL/6J mice injected with WT CAR-T cells were sacrificed on a weekly basis to harvest bone marrow mononuclear cells (BMMC) (Figure 3H). BMMCs collected from IL-2Ra KO and C57BL/6J mice per time point were used for isolating neutrophils stained with Annexin V (marker for apoptosis) as well as BrDU (marker for proliferation). The percentage of total mature neutrophils present in the bone marrow was also measured. IL-2Ra KO mice showed a significant increase in the rate of apoptosis (*P* < 0.0001) as indicated by an increase in Annexin V+ 7-AAD+ neutrophils, classified as late-stage apoptotic cells that peaked at week 3 after WT CAR-T (Figure 3I). There was no noticeable difference in apoptosis among neutrophils derived from WT CAR-T–treated C57BL/6J mice. Although a significant decrease in mature neutrophils was observed in both IL-2Ra KO and C57BL/6J mice (Figure 3J), only IL-2Ra KO mice had a significant increase in neutrophil apoptosis and reduced proliferation, confirmed by a decrease (*P* = 0.002) in BrDU+ neutrophils (Figure 3K).

### IFN-γ blockade aids recovery of CRS and neutropenia following CAR-T treatment.

**Figure 4:**
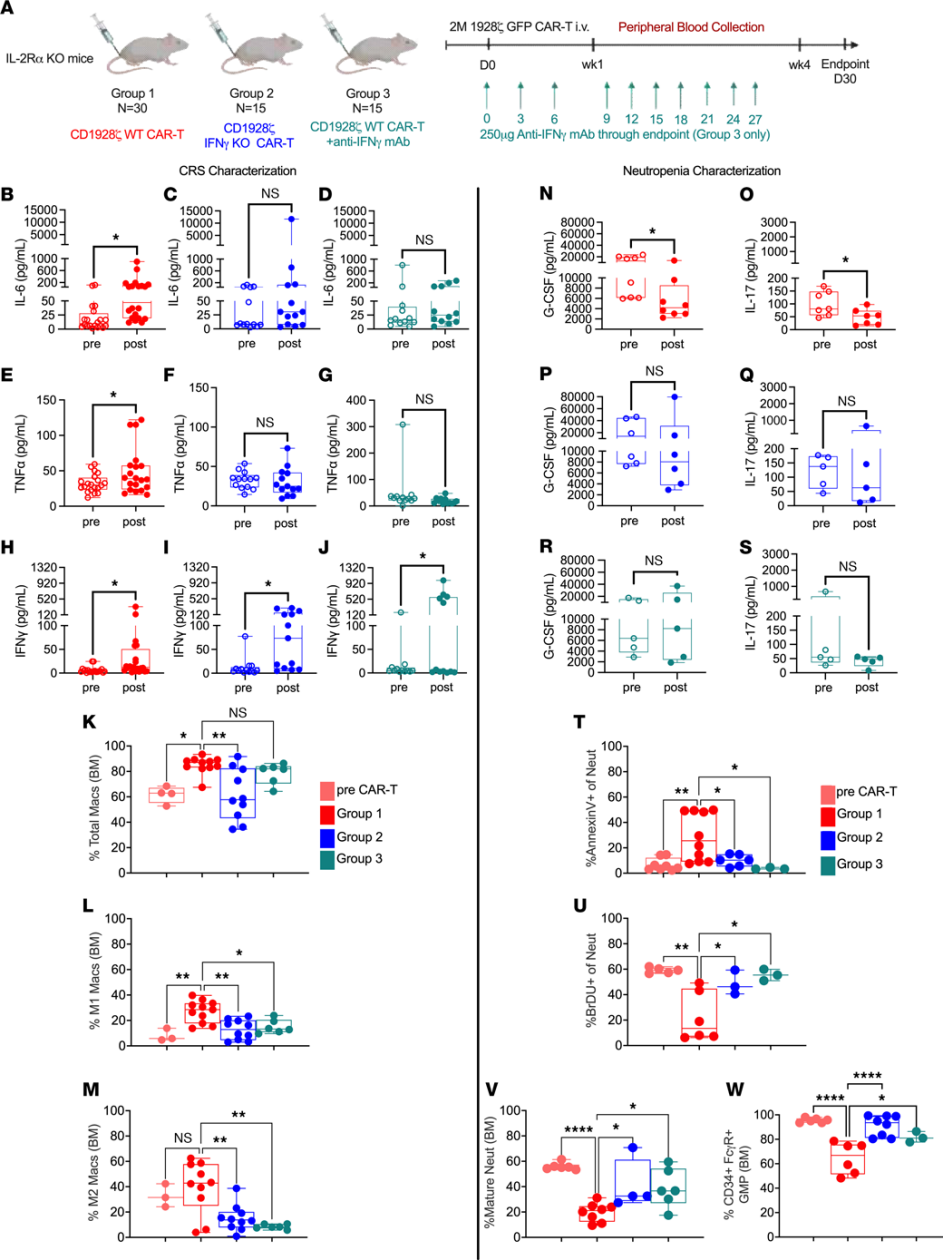
Effect of IFN-γ blockade on CRS and Neutrophils. (**A**) IL-2Ra KO mice treated with WT CAR-T (Group 1), or with IFN-γ KO CAR-T cells (Group 2) from IFN-γ KO mice or with anti-IFN-γ mAb (Group 3). Data are pooled from 2 independent experiments. (**B**–**J**) For CRS characterization, CRS-associated cytokines IL-6, TNF-α, and IFN-γ were analyzed in Group 1 (**B**, **E**, and **H**), Group 2 (**C**, **F**, and **I**), and Group 3 (**D**, **G**, and **J**), respectively. Pre and postcytokine levels are paired values taken from each mouse alive at week 4 per cohort (Group 1 *n* = 20, Group 2 *n* = 13, and Group 3 *n* = 12). (**K**–**M**) Flow cytometric analysis of macrophages used mice alive at week 4 from groups 1–3 (Group 1: *n* = 11; 2: *n* = 10; 3: *n* = 6). The frequency of (**K**) total macrophages (Live+CD45+CD11b+F4/80+), (**L**) iNOS+M1-like macrophages (Live+CD45+CD11b+F4/80+ CD11c-CD86+MHCII+iNOS+), and (**M**) Arginase1+M2-like macrophages (Live+CD45+CD11b+F4/80 CD11c-+CD206+Arginase1+) are represented as a percent of CD45+ cells. (**N**–**S**) For characterizing neutrophils, cytokines G-CSF (**N**, **P**, and **R**) and IL-17A (**O**, **Q**, and **S**) were evaluated in groups 1–3. Paired cytokine values for IL-17A and G-CSF are from 1 experiment (Group 1, *n* = 8, Group 2 *n* = 6, Group 3 *n* = 5). (**T**–**V**) Flow cytometry of neutrophil apoptosis and proliferation used a subset of mice alive at week 4 for Group 1 (*n* = 10 for apoptosis and *n* = 6 for proliferation), Group 2 (*n* = 6 for apoptosis and *n* = 3 for proliferation) and Group 3 (*n* = 3 for apoptosis and proliferation). Bar plots represent percentage of (**T**) neutrophil apoptosis (Annexin V+ cells as a frequency of purified neutrophils) and (**U**) neutrophil proliferation (BrDU+ cells as a frequency of purified live neutrophils). (**V**) % Ly6G+CXCR2+ mature neutrophils (gated on Live+Lineage-CD11b+Gr1+ckit-CXCR4- BMMCs) was measured in mice alive at week 4 (Group 1: *n* = 8; 2: *n* = 4; 3: *n* = 6). (**W**) Bar plots for % GMP cells used mice alive at week 4 gating on Live+ckit+Sca1-FcgR+CD34+ BMMCs in groups 1–3 (Group 1: *n* = 6; 2: *n* = 8; 3: *n* = 3). Error bars represent SEM. *P < 0.05, **P < 0.01, and ***P < 0.001. Statistics for cytokine bar plots (**B**–**J** and **N**–**S**) used paired *t* test; for **K**–**M** and **T**–**W**, used Tukey’s multiple comparison test (using 1-way ANOVA).

Macrophages mediate CRS by releasing proinflammatory cytokines (33, 34). Blockade of IFN-γ inhibits macrophage activation caused during CRS ex-vivo (21, 35). Excess IFN-γ during inflammatory conditions has been shown to favor myelopoiesis over granulopoiesis (36, 37). However, the effect of IFN-γ blockade on neutropenia in the context of CAR-T–associated toxicity has not been characterized. Therefore, IL-2Ra KO mice administered with CAR-T cells were divided into 3 groups: Group 1 included mice treated with WT CAR-T, Group 2 included mice treated with CAR T cells derived from IFN-γ KO mice, and Group 3 mice were treated with WT CAR-T followed by anti–IFN-γ mAb (Figure 4A). Week 4 analysis measured CRS-associated cytokines (IL-6, TNF-α, and IFN-γ) and cytokines that regulate neutrophils (IL-17A and G-CSF). There were no observable differences between the activation (CD69 and Granzyme B) and exhaustion (PD1 and TIM3) status of IFN-γ KO CAR-T cells when compared with WT CAR-T (Supplemental Figure 4, A–E).

WT CAR-T–treated IL-2Ra KO mice (Group 1) reproduced elevation of IL-6 and TNF-α, but these cytokines were reduced in IFN-γ KO CAR-T and anti–IFN-γ mAb groups (Figure 4, B–G). The increase in IFN-γ after CAR-T treatment was observed again after IFN-γ mitigation, likely due to the TMDD effect in Group 3 and secretion of IFN-γ by other immune cells in Group 2 (Figure 4, H–J) (Supplemental Figure 5, A–G). Despite the presence of IFN-γ, lack of increase in CXCL9 (downstream marker of IFN-γ activity) in Groups 2 and 3 suggests functional inhibition of downstream IFN-γ activity (Supplemental Figure 5, H–J). Analysis of BMDMs corroborated the significance of IFN-γ blockade to alleviate CRS. WT CAR-T administration in Group 1 increased the total macrophage population (F4/80+CD11b+) (*P* = 0.024) (Figure 4K). These BMDMs (from Group 1) were distinctly polarized toward the M1-like phenotype (CD86+MHCII+iNOS+) (*P* = 0.004), where iNOS expression is associated with CRS (Figure 4L), as compared with the M2-like phenotype (CD206+Arginase1+) (Figure 4M). There was a significant reduction in M1-like macrophages with either mode of IFN-γ blockade (Figure 4L).

The elevation of cytokines in Group 1 coincided with a significant decline in cytokines associated with neutrophil survival, G-CSF (*P* = 0.019) (Figure 4N), and IL-17A (*P* = 0.030) (Figure 4O). IL-17A stimulates fibroblasts that release G-CSF to activate the transcription factors such as Gfi1 and CEBPa, crucial for granulopoiesis (38–43). IFN-γ inhibition supported a recovery of G-CSF and IL-17A levels to baseline in Group 2 (Figure 4, P and Q) and Group 3 (Figure 4, R and S). Endpoint analysis revealed a significant reduction in the percentage of bone marrow–derived apoptotic neutrophils (Figure 4T) with IFN-γ blockade in Group 2 and Group 3. This corresponded with a significant increase in neutrophil proliferation (Figure 4U) in Group 2 (*P* = 0.048) and Group 3 (*P* = 0.012). The inhibition of mature neutrophils (Figure 4V) in Group 1 (*P* < 0.0001) showed a significant recovery with IFN-γ mitigation (Group 2, *P* = 0.04, Group 3, *P* = 0.034). The increase in neutrophil apoptosis and poor neutrophil recovery in WT CAR-T–treated IL-2Ra KO mice prompted an investigation into the impact of cytokines on granulocyte-monocyte progenitors (GMP) or granulocyte progenitors (GP). GMP and GP cells were FACS sorted using lineage-depleted bone marrow cells of IL-2Ra KO mice 4 weeks after treatment with WT CAR-T cells or IFN-γ KO CAR-T cells. The sorted GMP/GP cells were cultured ex-vivo and their colony formation potential was measured (Supplemental Figure 6A). WT CAR-T–treated IL-2Ra KO mice had significantly dampened GMP/GP colony formation not observed in C57BL/6J mice or IL-2Ra KO mice treated with IFN-γ KO CAR-T cells (Supplemental Figure 6, B–D). Flow cytometric analysis of GMPs shown as percentage CD34+FcgR+ cells in bone marrow confirmed a significant drop in GMP population from WT CAR-T–treated IL-2Ra KO mice (Figure 4W, *P* < 0.0001), while deletion of IFN-γ in CAR T cells or IFN-γ blockade restored neutrophil progenitors to pre-CAR-T levels shown in Group 2 (*P* < 0.0001) and Group 3 (*P* = 0.0484).

### IFN-γ Modulation of the Th1/Th17 Axis Drives CRS and Neutropenia.

**Figure 5:**
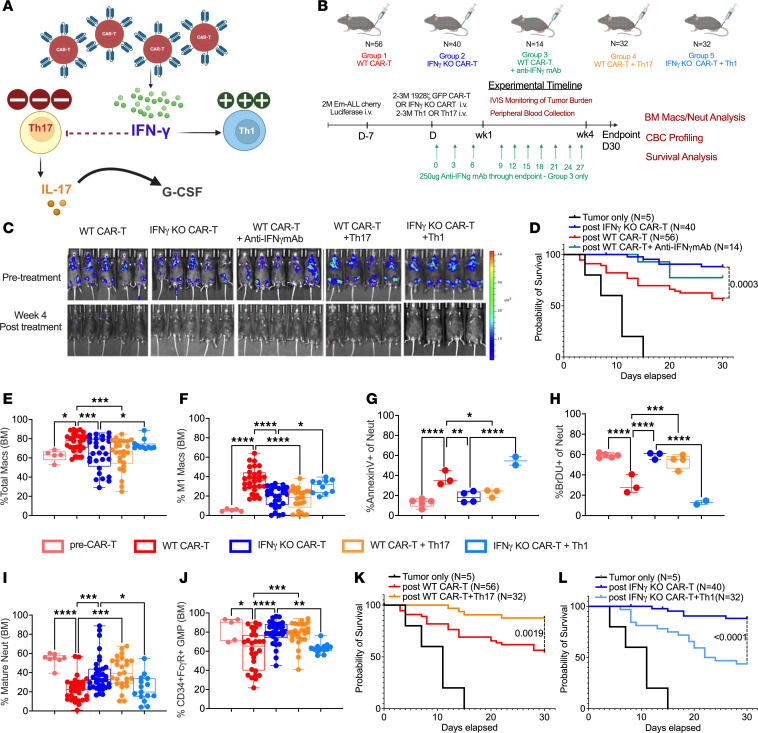
The impact of IFN-γ blockade on the Th1/Th17 axis after CAR-T administration. (**A**) Representation of proposed mechanism for the role of IFN-γ in regulating Th1/Th17 axis during CRS and neutropenia. (**B**) IL-2Ra KO mice bearing luciferase-expressing Em-ALL tumors administered with WT CAR-T (Group1), IFN-γ KO CAR-T (Group 2), WT CAR-T treated with anti-IFN-γ mAb (Group 3), WT CAR-T + Th17 cells (Group 4), and IFN-γ KO CAR-T with Th1 cells (Group 5). A control group included mice injected with tumor only. Mice were imaged pretreatment and at week 4 (endpoint) followed by analysis of CBC, neutropenia, and CRS. Data in groups 1, 2, 4, and 5 are pooled from 3 independent experiments. Group 3 and tumor only are from 1 of the pooled experiments, so no statistics for these groups are included. (**C**) IVIS images showing tumor burden pretreatment and at endpoint. (**D**) Difference in Kaplan-Meier OS of mice between groups 1, 2,and 3. % Total macrophages (Live+CD45+CD11b+F4/80+) (**E**) and % iNOS+M1-like macrophages (Live+CD45+CD11b+F4/80+CD11c-CD86+MHCII+iNOS+) (**F**) were compared. (**G**) % Annexin V+ apoptotic cells as a frequency of neutrophils (kit-based purification), (**H**) % BrDU+ cells as a frequency of live neutrophils (kit-based purification), and (**I**) % Ly6G+CXCR2+ mature neutrophils (gated on Live+Lineage-CD11b+Gr1+ckit-CXCR4- BMMCs) for assessing neutrophil recovery. (**J**) % GMP cells were determined by gating on Live+ckit+Sca1-FcgR+CD34+ BMMCs. (**K–L**) Difference in Kaplan-Meier OS between groups 1 and 4 (**K**) and groups 2 and 5 (**L**). Mice used in groups 1, 2, and tumor only are the same in **D**, **K**, and **L**. Mice alive at endpoint were used to determine macrophages (Group 1: *n* = 29; 2: *n* = 28; 4: *n* = 26; 5: *n* = 10), mature neutrophils (Group 1: *n* = 31; 2: *n* = 34; 4: *n* = 26; 5: *n* = 14), and GMPs (Group 1: *n* = 28; 2: *n* = 29; 4: *n* = 26; 5: *n* = 13) in **E**, **F**, **I**, and **J**. Annexin V and BrDU staining was done using mice from groups 1 (*n* = 3 for Annexin V and BrDU), 2 (*n* = 4 for Annexin V and *n* = 3 for BrDU), 4 (*n* = 3 for Annexin V and *n* = 4 for BrDU), and 5 (*n* = 2 for Annexin V and BrDU) in **G** and **H**. Error bars represent SEM. *P < 0.05, **P < 0.01, and ***P < 0.001. *P* values for Kaplan-Meier survival curves **D**, **K**, and **L** were generated using Log-rank (Mantel-Cox) test. Statistics for box plots (**E**–**J**) generated using Tukey’s multiple comparison test (1-way ANOVA).

To delineate the influence of IFN-γ on Th1/Th17 balance and neutrophil homeostasis (Figure 5A), naive CD4+ CD62Lhi CD44low cells were isolated from C57BL/6J (B6) mice and confirmed to have no expression of the Th1 cytokine IFN-γ or the Th17 cytokine IL-17A (Supplemental Figure 7, A and B). Isolated naive CD4+ T cells were cultured with Th1- or Th17-inducing cytokines followed by coculture with WT CD1928z CAR-T cells or IFN-γ KO CD1928z CAR-T cells (Supplemental Figure 7C). Differentiation of Th0 to Th17 was 38.1% when cultured without CAR T cells (Supplemental Figure 7D). When these cells were cocultured with WT CD1928z CAR-T cells, differentiation to Th17 dropped to 1.9%. Coculture with IFN-γ KO CAR-T cells supported 17.3% Th0 cells to differentiate into Th17 (Supplemental Figure 7D). Without CAR T cells, 85% Th0 cells differentiated into Th1, which dropped to 59.75% in the presence of WT CD1928z CAR-T cells and 8.29% in presence of IFN-γ KO CAR-T cells.

To further interrogate the role of the Th1/Th17 axis on CAR T function, IL-2Ra KO mice were inoculated with syngeneic Em-ALL that expressed luciferase (Figure 5B). A week later, mice with established tumors were divided into 5 groups. In addition to Group 1 (WT CAR-T), Group 2 (IFN-γ KO CAR-T), and Group 3 (WT CAR-T +anti- IFN-γ mAb), Group 4 included WT CAR-T and Th17 cells that were adoptively transferred at a 1:1 ratio. Group 5 mice were administered IFN-γ KO CAR-T followed by an equal number of Th1 cells. CAR-T expansion was unaltered in presence of Th17 or Th1 (Supplemental Figure 8, A and B). As reported by Bailey et al. (21), we confirm that IFN-γ blockade does not alter the antitumor efficacy of CD1928z CAR-T cells (Figure 5C). Moreover, IFN-γ blockade improved the survival of mice after CAR-T infusion (*P* = 0.0003 for Group 1 versus Group 2) (Figure 5D). We also noted total and M1-like macrophages are significantly reduced by IFN-γ blockade (Figure 5, E and F). There were no significant changes in the frequency of M2-like macrophages in Groups 1, 2, and 3 (Supplemental Figure 8C). However, WT CAR-T cells induced iNOS in M2-like macrophages that was reduced with IFN-γ blockade (Group 2) (Supplemental Figure 8D). These observations reproduce our recent report showing that CAR-T induced their own suppression by upregulating iNOS in M2-like macrophages, which could be reversed when blocking IFN-γ (44). We also noted increases in apoptotic neutrophils and decreases in neutrophil proliferation, maturation and GMP frequency, which could all be reversed by IFN-γ attenuation (Figure 5, G–J).

We hypothesized that Th0 to Th1 differentiation caused by IFN-γ released during CAR-T treatment results in an inadequate reserve of Th0 cells for Th17 differentiation. This is confirmed by addition of Th17 cells to WT CAR-T treated mice (Group 4), which displayed reduced neutrophil apoptosis, enhanced neutrophil proliferation, and maturation (Figure 5, G–I). Th17 adoptive transfer also led to increase in GMPs (Group 1 vs Group 4: *P* = 0.0002) (Figure 5J). This improvement of BM neutrophil homeostasis due to Th17 adoptive transfer increased neutrophil concentration in peripheral blood compared with WT CAR-T alone (Supplemental Figure 8, E and G). Moreover, Th17 cells reduced total and M1-like macrophages (Figure 5, E and F) and significantly improved the survival of WT CAR-T–treated mice (Figure 5K). We hypothesized that IFN-γ released by CAR-T cells increases Th1 differentiation and M1-like macrophages, which was validated through Group 5, showing adoptive transfer of Th1 cells reversed the benefit of IFN-γ blockade on toxicities by triggering polarization of M1-like macrophages (Group 2 versus Group 5: *P* = 0.049) (Figure 5F). Th1 adoptive transfer also led to an increase in apoptotic neutrophils and a decrease in proliferative and mature neutrophils compared with the IFN-γ KO CAR-T group (Figure 5, G–I). IFN-γ KO CAR-T treatment showed no difference in peripheral blood neutrophils (Supplemental Figure 8F), whereas adoptive transfer of Th1 resulted in a significant drop in neutrophil concentration (Supplemental Figure 8H). Reestablishment of CRS and neutropenia in IFN-γ KO CAR-T–treated mice infused with Th1 cells resulted in poor survival (*P* < 0.0001) (Figure 5L). Since addition of Th1 preserved the expansion and efficacy of IFN-γ–KO CAR-T (Figure 5C and Supplemental Figure 8B), the cause of death contributing to poor survival in Group 5 is most likely due to rapid onset of CRS and not the tumor.

### Single-cell characterization of bone marrow neutrophils and macrophages in IL-2Ra KO mice during CRS and neutropenia.

**Figure 6:**
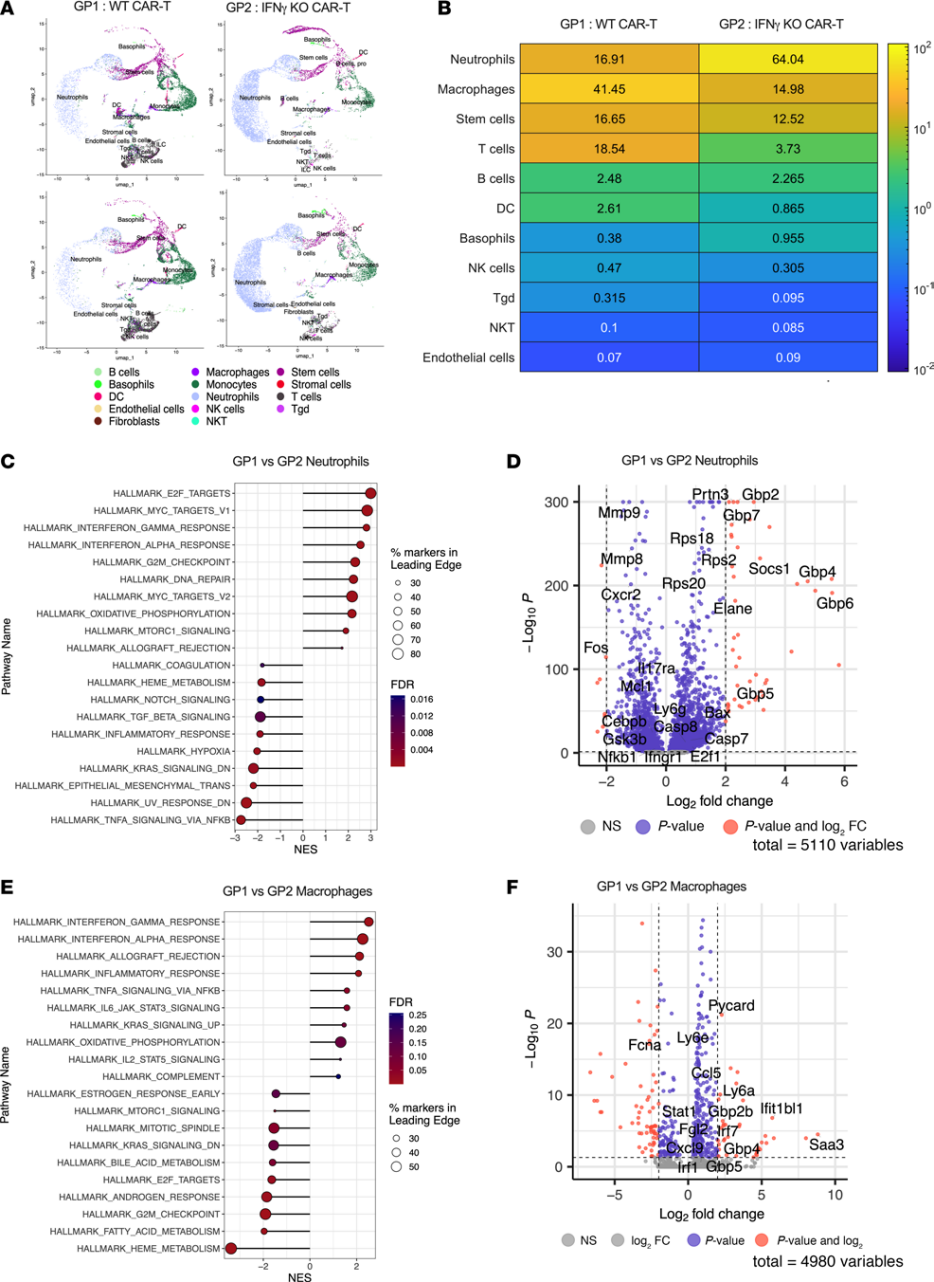
Utilizing single cell–sequencing to identify phenotypic differences in bone marrow neutrophils and macrophages from WT CAR-T and IFN-γ KO CAR-T–treated mice. (**A**) UMAPs representing cell types present in bone marrow samples of WT CAR-T treated (Group 1 or GP 1) and IFN-γ KO CAR-T treated (Group 2 or GP 2) samples. Each plot represents an individual mouse with 2 mice per group. (**B**) Heat map displaying the frequency of immune and nonimmune cells in GP 1 and GP 2. Computed values are an average of 2 mice per Group. (**C**) Gene set enrichment analysis (GSEA) showing the various Pathways from Hallmark dataset that were enriched in the comparison of Neutrophils between GP 1 and GP 2. (**D**) Volcano plots highlighting the most differentially expressed genes in the Neutrophils from GP 1 and GP 2. (**E**) GSEA showing various Pathways from Hallmark dataset that were enriched in the comparison of Macrophages between GP 1 and 2. (**F**) Volcano plots highlighting the most differentially expressed genes in the Macrophages from GP 1 and 2.

We performed single-cell sequencing to compare differences in neutrophils and macrophages from BMMCs isolated at week 4 from Group 1 (WT CAR-T, *n* = 2), Group 2 (IFN-g KO CAR-T, *n* = 2), Group 4 (WT CAR-T+Th17, *n* = 2), and Group 5 (IFN-g KO CAR-T +Th1, *n* = 2). We used uniform manifold approximation and projection (UMAP) (45) to represent every cell in a 2-dimensional embedding, which facilitates cell type identification based on shared gene expression markers in clusters of cells (informed by data from the Immunological Genome Project, ImmGen (46)). UMAP plots for each sample showcase different cell types, their respective numbers, and their molecular heterogeneity, with nearly 70% of BMMCs in IL-2Ra KO mice being either neutrophils or macrophages/monocytes (Figure 6A and Supplemental Figure 9A). Frequency of neutrophils are improved from Group 1 (16.9%, Standard deviation ‘SD’=3.8%) to Group 2 (64%, SD=5.3%) (*P* = 0.009) owing to IFN-γ attenuation (Figure 6B). Similarly, Th17 administration in Group 4 increased neutrophils (63.2%, SD=5.7%) (*P* = 0.011) (Supplemental Figure 9B). Conversely, Th1 administration in Group 5 slightly reduced neutrophils (50.6%, SD=12.3%) (*P* = 0.293) (Supplemental Figure 9B) compared with Group 2 (IFN-γ KO CAR-T). We also note macrophages in Group 1 (41.45%, SD=1.8%) were reduced by IFN-γ mitigation in Groups 2 (15%, SD=1.2%) (*P* = 0.003) and 4 (17.9%, SD=2.9%) (*P* = 0.008) (Supplemental Figure 9B). Th1 administration in Group 5 (19.2%, SD=10.1%) yielded a small increase (*P* = 0.62) compared with IFN-γ KO CAR-T (Supplemental Figure 9B). In spite of small sample size of single-cell sequencing, we see statistical significance in cell type frequencies for all comparisons except Group 5 versus Group 2. However, standard deviations within each group indicate low variability between the samples in a given group.

Gene set enrichment analysis (GSEA) (45) of cancer hallmarks pathways in neutrophils showed upregulation of IFN-γ response genes in Group 1 compared with Group 2 (Figure 6C), which is confirmed by higher expression of IFN-γ–inducible guanylate binding proteins (Gbp2, Gbp4, Gbp6, and Gbp7) (47) (Figure 6D). This correlated with a downregulation of transcription factors involved in neutrophil activation and emergency granulopoiesis (Nfkb1, Gsk3b, and Cebpb) (48–50). Furthermore, overexpression of Prtn3, previously observed in apoptotic neutrophils (51), in addition to degradation of c-Fos (52) are indicative of increased apoptosis being triggered in Group 1 neutrophils. An upregulation of cell cycle gene sets involving E2F is attributed to overexpression of E2f1 and MYC due to overexpression of Casp8 and Bax (Figure 6, C and D) that drive MYC-induced apoptosis (53–55). Upregulation of E2f1 is associated with overexpression of Casp7 and underexpression of Mcl1 in neutrophils of Group 1 compared with Group 2, which support E2f1’s role in apoptosis (56–59). However, Group 2 neutrophils are enriched for heme metabolism, hypoxia, and TNF-α signaling via NFKB (Figure 6C), which promote neutrophil survival (60–62). We also note that Group 1 macrophages are enriched for IFN-γ and IFN-α response pathways compared with Group 2 (Figure 6E), which is attributed to upregulation of Ccl5, Stat1, and Fgl2 genes involved in M1-like macrophage polarization via the Cxcl9/Cxl10 axis (Figure 6F) (63–65) (44). We observed over expression of IFN regulatory factors, IRF1 (induced by IFN-γ) and IRF7 (induced by IFN-α), which, in turn, aid in the production of iNOS (66–68). Collectively, these analyses offer insight into putative molecular mechanisms involved in excess M1-like macrophage polarization and subsequent activation of neutrophil apoptosis during CRS and neutropenia cooccurrence. These molecular signatures are also seen in Group 1 (WT CAR-T) versus Group 4 (Th17 adoptive transfer) and Group 2 (IFN-γ KO CAR-T) versus Group 5 (Th1 adoptive transfer) comparisons (Supplemental Figure 10, A–H).

### Association of Th1/Th17 cytokine levels in human serum with cooccurrence of CRS and neutropenia in CAR-T–treated patients.

**Figure 7:**
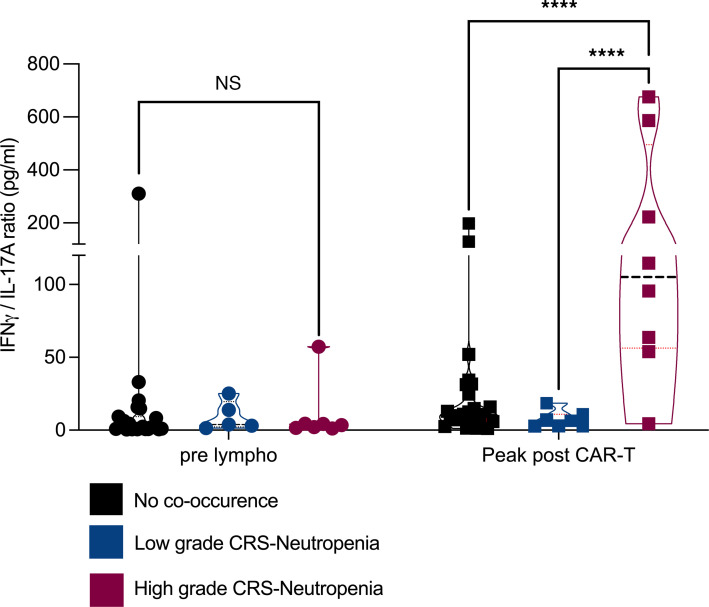
Determining the association of Th1/Th17 balance with CRS Neutropenia cooccurrence in patients. Ratios of IFN-γ to IL-17A (IFN-γ/IL-17A) were calculated for 43 patients with hematological malignancies. Individual levels of IFN-γ as well as IL-17A at prelymphodepletion (prelympho: 6 days before CAR-T infusion) and peak post-CAR-T infusion (defined as the highest cytokine value from postinfusion serum collected on days 1, 7, and 11) were used to generate the ratio of IFN-γ to IL-17A. Violin plots represent 3 different groups where the plots in black represent patients that did not develop cooccurrence of CRS and neutropenia (*n* = 28), blue violin plots represent patients with a mild case of CRS and neutropenia cooccurrence (*n* = 7), and red violin plots represent patients with a severe case of CRS and neutropenia cooccurrence (*n* = 8). *P < 0.05, **P < 0.01, ***P < 0.001 were derived from unpaired *t* tests.

We investigated if Th1/Th17 balance is associated with CAR T cell toxicities in 43 patients treated at the Roswell Park Comprehensive Cancer Center (Supplemental Table 1). These patients were graded for CRS based on ASTCT guidelines (69). We utilized a neutropenia classification by Rejeski et al (70) (Supplemental Table 2) that categorizes neutropenia based on rate of recovery (Quick, Intermittent, and Aplastic) and another based on severity (Protracted, Profound, Prolonged) per ASCO/IDSA consensus guidelines (71). To determine the role of Th1/Th17 imbalance in driving cooccurrence of CRS and neutropenia in 43 CAR-T–treated patients with hematological malignancies, we split the patients into 3 groups: no cooccurrence of CRS neutropenia, low-grade CRS neutropenia, and high-grade CRSneutropenia. Low-grade CRS neutropenia was defined by cooccurrence of neutropenia and grade 1 CRS. High-grade CRS neutropenia was defined by grade 2 or higher-grade CRS along with prolonged or aplastic neutropenia (severe/high-grade neutropenia). We considered grade 2 CRS as high grade since it requires advanced clinical intervention (13). Eight patients experienced high-grade CRS neutropenia, 7 patients experienced low-grade CRSneutropenia, and the remaining 28 patients experienced no cooccurrence of CRS neutropenia. We measured the ratio of IFN-γ (induced by Th1 cells) to IL-17A (induced by Th17 cells) cytokine levels at prelymphodepletion and peak-postinfusion. A significant increase in IFN-γ–to–IL-17A ratio was noted between high-grade CRS neutropenia and no cooccurrence (*P* < 0.0001) as well as low-grade CRS neutropenia (*P* < 0.0001) groups at peak-postinfusion (Figure 7A). The smaller ratio of IFN-γ to IL-17A in low-grade CRS neutropenia compared with high-grade CRS neutropenia may be attributed to the success of CRS intervention strategies. In conclusion, this data is consistent with our in vivo studies, where we observed cooccurrence of CRS neutropenia associated with Th1/Th17 balance.

## Discussion

The advent of CAR-T therapy has greatly improved outcomes for patients with hematological malignancies; however, a subset of patients continue to experience toxicities such as CRS, ICANS, and ICAHT (1). Rejeski et al show that 51% of nonrelapse patient mortality (NRM) is due to infections attributed to cytopenias (12). There are currently no preclinical in vivo models that recapitulate cytopenias. While mouse models for CRS exist (33, 34) they are not immune competent and not ideal to study cytopenias. We employed an IL-2Ra KO in vivo model to recapitulate CRS in Em-ALL tumor-bearing mice and observed a significant increase in proinflammatory cytokines and death from CRS. It is unknown if other Treg-deficient models will recapitulate CRS, since IL-2Ra also has a Treg-independent role in moderating inflammation (72, 73). Nontumor-bearing IL-2Ra KO mice treated with WT CAR-T also exhibited CRS, which anti–IL-6R or anti–IFN-γ mAb alleviated. The predominant manifestation of ICAHT is prolonged neutropenia characterized by a hypocellular bone marrow that is nonresponsive to growth factors and is without effective treatment (6). ICAHT is posited to be related to cytokines, since clinically, CRS and neutropenia cooccur, and ICAHT after CAR-T cell therapy is categorically different than the neutropenia observed from chemotherapy alone (6, 7, 9). Similar to patients, CAR-T treatment in the IL-2Ra KO model resulted in prolonged reduction of neutrophils, while lymphodepletion-induced neutropenia had rapid recovery. Prolonged neutrophil reduction associated with WT CAR-T treatment was shown to coincide with a disruption of bone marrow neutrophil homeostasis. This was accompanied by an increase in proinflammatory M1-like macrophages. The IL-2Ra KO mouse model does not recapitulate thrombocytopenia, which is also frequently observed in patients (11). The model-specific differential impact on neutrophils and platelets due to proinflammatory cytokines involved in CRS may be the cause for the absence of thrombocytopenia in our model, highlighting the need for more robust models accounting for thrombocytopenia.

We investigated the role of IFN-γ blockade on CAR-T–treated IL-2Ra KO mice in 2 ways: (a) by combining WT CAR-T with anti–IFN-γ mAb resulting in a systemic inhibition of IFN-γ and (b) by employing IFN-γ KO CAR-T for a more targeted effect. Using either form of IFN-γ blockade, we noted an alleviation of CRS and neutropenia, thereby implicating excess IFN-γ produced by rapidly expanding CAR-T cells as a key mediator of these toxicities. Most notably, we observed no significant differences in CAR-T efficacy with IFN-γ blockade, and between both systemic (using anti–IFN-γ mAb) and targeted (IFN-γ KO CAR-T) blockade of IFN-γ, which is consistent with findings from a recent study involving hematological malignancies (21). CAR-T–associated CRS is characterized by an increase in cytokines IFN-γ and TNF-α, while neutropenia is characterized by a decrease in IL-17A and G-CSF. Th17 cells produce IL-17, which regulates G-CSF (74), which is responsible for granulopoiesis, while Th1 cells produce IFN-γ (75), which supports proinflammatory M1-like macrophages (76), leading to CRS. Both Th1 and Th17 cells originate from naive CD4+ T cells, which differentiate into Th1 cells in the presence of IL-12 and IFN-γ (77), while Th17 differentiation is supported by TGF-β and IL-6 (78). Tregs play a crucial role in balancing the Th1-Th17 axis by producing TGF-β, aiding in Th17 differentiation (79) and IL-10, which suppresses antigen presenting cells (APC) from releasing IL-12 (80). IL-2Ra KO in mice leads to Treg dysfunction, resulting in breakdown of such negative feedback loops. In the absence of Tregs, IFN-γ released by CD8+ T cells induce APCs to produce IL-12, leading to Th1 differentiation, more IFN-γ secretion, and a positive feedback loop (81). The production of IFN-γ by multiple sources shown in Supplemental Figure 11 would explain the pronounced elevation in IFN-γ compared with other CRS cytokines in our model. Systemic and targeted IFN-γ inhibition prevent uncontrolled Th1 differentiation and may temporarily reinstate homeostatic immune function and cytokine balance.

Using single-cell RNA-seq we characterized bone marrow neutrophils and macrophages, which revealed upregulation of apoptotic signaling in neutrophils and overexpression of markers associated with M1-like polarization in macrophages in IL-2Ra KO mice treated with WT CAR-T cells. The role of Th1/Th17 imbalance driving CRS and neutropenia is verified with single-cell RNA-seq, where similar molecular characteristics were observed after adoptive transfer of Th1 cells with IFN-γ KO CAR-T compared with WT CAR-T cells alone and IFN-γ KO CAR-T treatment compared with adoptive transfer of Th17 cells. Moreover, the role of Th1/Th17 imbalance in the context of cooccurrence of CRS neutropenia is shown using patient cytokines. Peripheral blood samples of patients treated with CAR-T show an increase in IFN-γ–to–IL-17A ratio between high-grade CRS neutropenia and no cooccurrence of CRS neutropenia. This is also confirmed by a recent study that shows a similar decrease in IL-17A to be associated with CRS and ICANS (82).

Our work identifies the downstream mediators of IFN-γ responsible for CRS and neutropenia that lead to adverse patient outcomes. IFN-γ has a pleiotropic role on antitumor immunity, so we speculate that its blockade during CAR-T therapy could have different effects on toxicities and efficacy based on when and how it is blocked. For example, we recently demonstrate that IFN-γ secreted by CAR-T cells induces iNOS in M2-like macrophages that lead to CAR-T suppression and that IFN-γ blockade improves CAR-T tumor killing (44). In contrast, a study by Tang et. al. shows that lack of IFN-γ maintains V-domain Ig suppressor of T-cell activation (VISTA) that inhibits tumor killing efficacy and persistence of CD19-CAR-T cells (83). This pleiotropic effect of IFN-γ suggests that its blockade could have different effects. Therefore, trials such as NCT06550141 will be critical to inform our understanding of the impact of IFN-γ blockade on toxicities induced by CAR-T cell therapy.

## Methods

### Sex as a biological variable.

We employed both male and female individuals in our human and animal studies; however, biological sex was not considered a variable in our analysis.

### Mice.

All animal studies were conducted per protocols approved by the Institutional animal care and use committee (IACUC) of the Roswell Park and Moffitt Comprehensive Cancer Centers. IL2-Ra KO mice (B6;129S4-IL-2Ratm1Dw/J, RRID:IMSR_JAX:002462) were obtained from JAX lab and bred in-house. IL-2Ra KO mice were created by replacing exons 2 and 3 in IL-2Ra gene with a neomycin resistance cassette (73). Young IL-2Ra KO mice displayed normal T and B cell development, however, their systemic knockout of IL-2Ra may trigger inflammatory disorders such as splenomegaly, lymphadenopathy and Inflammatory bowel disease at a later stage in their lives (84). Mice positive for neomycin allele and negative for WT allele were confirmed by genotyping as IL-2Ra KO and used for in vivo studies. C57BL/6J mice (RRID:IMSR_JAX:000664) were purchased by JAX laboratory and used as controls. Thy 1.1 (B6.PL-Thy1a/CyJ, RRID:IMSR_JAX:000406) were used as donors for the generation of WT Thy 1.1 CD19-28z CAR-T cells while IFN-γ KO mice (B6.129S7-IFN-gtm1Ts/J, RRID: IMSR_JAX:002287) were used as donors for the generation of IFN-γ KO 19-28z CAR-T cells. WT CAR-T and IFN-γ KO CAR-T cells express IL-2Ra. Both male and female mice between 8 to 12 weeks old were used in experiments.

### Cell lines.

Eμ-ALL cherry Luciferase-expressing cells are a murine syngeneic malignant B cell line generated in house and maintained as described (20). All cell culture reagents were purchased from Thermo Fisher Scientific. Mouse CAR-T cells, Naive CD4+ T cells, Th1, and Th17 cells were cultured in RPMI-1640 supplemented with 10% FBS, 2 mmol/L L-glutamine, penicillin (100 U/mL), streptomycin (100 μg/mL) 2 mmol/L sodium pyruvate, 1 × nonessential amino acids, 10 mmol/L HEPES and 55 μmol/L β-mercaptoethanol. Universal Mycoplasma Detection Kit (ATCC) was used to test cell lines used in various experiments for mycoplasma.

### Genetic constructs and CAR-T cell generation.

The CD1928z CAR-T construct has been described (20, 85, 86). The construct was used for retroviral production and T cell transduction per our established protocol (87). Transduction efficiency was confirmed as a percentage of total GFP+ or Cherry+ T cells (listed in graphical representations or legends in main and Supplemental Figures), as the CARs include a fluorescent reporter protein.

### In vivo studies.

IL-2Ra KO or C57BL/6J mice were injected intravenously (i.v.) with 1 million (M) to 2 M murine syngeneic Eμ-ALL cells expressing luciferase. A week after tumor establishment, mice were administered intraperitoneally (i.p.) with 200 to 300 mg/ kg of cyclophosphamide (Sigma-Aldrich) followed by infusion of 1 M to 3 M 19–28z CAR-T cells. For Th1/Th17 adoptive transfer studies, Th1 or Th17 cells were injected at an equal ratio to CAR-T infusion. Tumor-bearing mice were randomized prior to being assigned to different experimental groups.

For cytokine analysis, serum was collected from peripheral blood through the submandibular vein. Cytokines were measured using 25 mL serum on a Luminex 100 system with a mouse luminex assay kit (R&D Systems) or Ella with a Simple Plex Assay Kit (Biotechne) according to manufacturer’s instructions.

For histopathology, mouse spleen and Lymph node tissues were collected at endpoint and stained using anti-B220 (CD45R) and anti-CD3 Antibodies. H&E staining was performed for spleen, lymph node, lung and liver sections and examined using Leica light microscopy.

For analysis of peripheral blood, samples were subjected to ACK lysis buffer (GIBCO, Thermofisher scientific) for 15 minutes at room temperature followed by staining with anti-CD19 (BD Biosciences) to measure levels of B cells and CD3 as well as Thy1.1 marker to detect levels of Thy1.1+GFP+ CAR-T cells in peripheral blood (PBL) (Supplemental Table 3).

For CRS mitigation using blockade of IL-6R and IFN-γ, IL-2Ra KO and C57BL/6J mice were treated intraperitoneally with either 12.5 mg/kg (250–300 mg per mouse weighing 20 g) of murine anti–IL-6R mAb (MP5-20F3; BioXcell) or 250 mg (per mouse weighing 20 g) of murine anti–IFN-γ mAb (Clone XMG1.2, Bioxcell) or Isotype control (Rat IgG1, clone HRPN, BioXcell), as described (88). Treatments were repeated twice weekly until endpoint.

For Treg adoptive transfer studies, murine CD4+CD25+Foxp3+ Tregs were isolated using a kit (STEMCELL) from the spleens of C57BL/6J mice (RRID:IMSR_JAX:000664), per the manufacturer’s instructions. Purified cells were resuspended in 1X PBS (100 L per mouse) and injected into IL-2Ra KO mice at a dose of 0.25 M Tregs or 1 M Tregs. Two million WT CAR-T cells were infused 2 days after transfer of Tregs.

For complete blood profiling to assess neutrophil concentration, 50 mL of peripheral blood was collected per mouse. The blood samples were measured using ProCyte Dx Hematology Analyzer.

For BrDU staining, neutrophils were isolated from bone marrow of IL-2Ra KO or C57BL/6J mice pre and postinfusion. Cells were counted and processed using the instructions provided by the kit manufacturer (BD Pharmingen; RRID: AB_2617060). Briefly, cells were stained with viability dye (Zombie NIR, Biolegend) and fixed and permeabilized using cytofix/cytoperm buffer for 30 minutes. The cells were then treated with DNAse (1 mg/mL) and incubated for an hour at 37°C followed by staining with anti-BrDU (BD Phramingen) at 1:50 dilution.

For Annexin V staining, isolated neutrophils were counted and washed twice with 1X Dulbecco’s phosphate buffered saline (GIBCO) per manufacturer instructions (BD Phramingen; RRID: AB_2869265). The cells were resuspended in 1X Annexin V binding buffer and stained using 5 mL anti-Annexin V as well as 5 mL 7-AAD for 15 minutes at room temperature in the dark. Cathepsin-treated cells were used as positive control. Stained samples were acquired using Cytek Aurora within 1 hour of preparation.

For neutrophil maturation analysis, 1 M total BMMC per sample were stained for flow cytometry with Zombie NIR (20 minutes, room temperature), followed by anti-CD3, anti-Nk1.1, anti-ckit, anti-CD115, anti-Gr-1, anti-Ly6G, anti-CXCR2, anti-CXCR4 (all from Biolegend; anti-CD11b and anti-B220 (both from BD Biosciences) (30 minutes, 4°C). Mature Neutrophils were gated as Live+Lineage- (CD3-, B-, NK-) ckit-CD115-Gr1+CD11b+CXCR4-Ly6G+CXCR2+ based on a previous study (89) (All antibodies used are listed along with clone numbers in Supplemental Table 4).

For characterization of M1-like and M2-like bone marrow macrophages, 1 M total bone marrow mononuclear cells (BMMC) per sample were stained for flow cytometry with Zombie NIR, anti-CD45, anti-F4/80, anti-CD64, anti-Ly6G, anti-Ly6C, anti-CD115, anti-MHCII, anti-iNOS, anti-Siglec-F (all from Biolegend); anti-CD11b, anti-CD11c and anti-CD86, anti-CD206 (BD Biosciences) and anti-Arginase1 (eBioscience). Based on a previous study (90). Cells were fixed (30 minutes, 4°C) and permeabilized for intracellular staining using Foxp3/Transcription Factor Staining Buffer Set (eBioscience). The % Total macrophages were gated as CD45+CD11b+F4/80+, % M1-like bone marrow macrophages were gated as CD45+CD11b+F4/80+CD11c-MHCII+CD86+iNOS+ and % M2-like macrophages were gated as CD45+CD11b+F4/80+CD11c-CD206+Arginase1+ as a percent total of CD45+ cells (Supplemental Table 5). All samples were incubated with Anti-Mouse CD16/CD32 (mouse Fc Block, BD Biosciences) prior to surface staining to inhibit any nonspecific binding.

### Colony formation assay and granulocyte monocyte progenitor characterization.

The colony formation assay (CFU) assay was performed by culturing FACS-sorted granulocyte monocyte progenetor (GMP) and GP cells in Methocult media (STEMCELL). BMMCs were first subjected to lineage depletion (Miltenyi Biotec) followed by FACS sorting of GMP (ckit+Sca1-CD16/32 (FcgR)+CD34+Ly6C-Flt3-CD115low) and GP (ckit+Sca1-CD16/32(FcgR)+CD34+Ly6C+Flt3-CD115low) populations using BD FACS Aria based on a previous publication (91). Sorted cells were pooled using 2 (out of 4) mice per group, to represent 2 replicates per group and resuspended at a 10X concentration of 0.2–0.3 M cells in IMDM + 2% FBS. The cells were seeded at a density of 20–30,000 cells in 1.1 mL Methocult per 35 mm plate prepared in duplicates per group. The duplicates for each group were then placed inside a 100 mm plate with a third 35 mm plate filled with water. The plates were incubated for 7 days at 37°C and 5% CO_2_. Colonies formed were imaged using phase contrast microscope and counted at day 7 using an STEMvision Hematopoietic Colony Counter. For flow cytometric characterization of GMPs, BMMCs were stained using anti-CD3, anti-B220, anti-Nk1.1, anti-ckit, anti-CD16/32 (FcgR), anti-Ly6C, anti-CD115, anti-Sca1, anti-Flt3 (all from Biolegend) and anti-CD34 (BD Biosciences). % GMP cells were gated as ckit+Sca1-CD16/32 (FcgR)+CD34+based on the previous study (91) (Supplemental Table 6).

### In-vitro Th1/Th17 differentiation assay.

The differentiation protocol was set up as described (92). Naive CD4+ T cells isolated (Miltenyi biotec) were seeded in 24 well plates at a concentration of 0.5 M cells per mL. Th1 differentiation cytokines included IL-12 at 10 ng/mL (R&D systems), IL-2 at 200 IU/mL (Iovance Biotherapeutics) and anti-IL-4 at 1 mg/mL (Thermofisher). Th17 differentiation cytokines included IL-6 at 40ng/mL (R & D systems), TGFb at 3ng/mL (R & D systems), anti-IL-4 at 1 mg/mL, anti-IL-2 and anti-IFN-γ at 1 mg/mL (Thermofisher). The differentiated cells were cultured either alone or in presence of 0.5 M WT CAR-T cells or IFN-γ KO CAR-T cells respectively (in separate wells). On day 4, cells were incubated with PMA at 5ng/mL (Sigma-Aldrich) and Ionomycin at 1mg/mL (Sigma-Aldrich) for an hour at 37°C. This was followed by addition of 10mg/mL Brefeldin A (eBioscience) and incubation for 4 hours. Stimulated T cells were further stained for flow cytometric analysis using anti-CD4, anti-CD44, anti-CD62L, anti-IL17 and anti–IFN-γ (Biolegend) (Supplemental Table 7).

### scRNA-seq methods.

Single cells were isolated and then processed using the 10X Genomics Single Cell 3’ v3 kit according to the manufacturer’s instructions. Libraries were sequenced on the Illumina NovaSeq 6000 instrument (RRID:SCR_016387). Raw sequencing data were processed using Cell Ranger (CR) (v6.0.0) pipeline (RRID:SCR_017344) to generate fastq files. Fastq files were aligned and quantified generating feature-barcode count matrices. Gene-barcode matrices containing Unique Molecular Identifier (UMI) counts are filtered using CR’s cell detection algorithm. Downstream analyses were performed mainly using Seurat (v5.0.0) single-cell analysis R package (RRID:SCR_016341). Eight single-cell RNA-seq samples were individually read into a Seurat object (RRID:SCR_016341) to examine feature number, mitochondrial percentage, and read count distributions within each sample. Cells with less than 500 features or greater than 7,500 features or greater than 15% mitochondrial content were filtered out. After normalizing and finding variables features from individual samples, all samples are then integrated using FindIntegrationAnchors function. The resulting data would serve for visualization purposes. Separately, samples are merged in a SingleR (v1.6.1), a Bioconductor package (RRID:SCR_006442), was then used to annotate individual immune cell types using ImmGen reference database (RRID:SCR_021792) from celldex (v1.12.0) R package. Principal component analysis was used to detect and visualize highly variable genes. Using the RNA assay, data normalization and scaling were performed using Seurat’s (RRID:SCR_016341) SCTransform function regressing against mitochondrial percentage. The data were then dimension reduced via UMAP (RRID:SCR_018217) and clustered using Louvain algorithm for downstream visualization. Normalization, scaling, dimension reduction, clustering, and quantification were performed on specific cell populations (i.e., Neutrophils and Macrophages) to further detect subpopulation clusters based on their expression profiles. Differential gene expression analysis was performed using Seurat’s (RRID:SCR_016341) FindMarkers function on groups of interest.

Subsequent GSEA were performed on comparisons of interest using GSEApreranked procedure implemented by the clusterProfiler package (v4.6.2) (RRID:SCR_016884) using msigDB database found in the msigdbr (v7.5.1) package (RRID:SCR_022870). Mouse versions of Hallmark, C2, and C5-GO gene sets were used to perform pathway analyses. The avg_log2FC values (obtained from the FindMarkers results) are utilized as ranked values to perform pathway analyses using prerankedGSEA. Specific cell-level signatures scores are obtained using Seurat’s (RRID:SCR_016341) AddModuleScore function. The custom code generated for all single-cell sequencing analysis is available at https://github.com/ecg-rpcc/Davila_CarT_BoneMarrow; commit ID a503a4fb7bd74d73579f7437fb1ce1f42cfa673b.

### Patient samples and data collection.

We obtained serum samples from all 43 patients who underwent CAR-T cell therapy at the Roswell Park Comprehensive Cancer Center between 14^th^ October, 2019 and 2^nd^ October, 2023; i.e., there was no attrition. The patients received standard-of-care CAR-T products and there were no inclusion or exclusion criteria applied during sample collection. Serum samples were collected at the following time points: prelymphodepletion (within 10 days prior to CAR-T infusion), Day 0 (pre-CAR-T), Day 1, Day 7, and Day 14 (post-CAR-T). Neutropenia classification followed criteria from a previous study (69). Prolonged neutropenia was defined as an absolute neutrophil count (ANC) < 1000 cells/μL, measured at ≥ 21 days after CAR-T cell infusion. Aplastic neutropenia was characterized by severe neutropenia (ANC < 500 cells/μL) lasting ≥ 14 days.

### Statistics.

All Statistical evaluations were performed using GraphPad Prism 10 software (GraphPad Software, La Jolla, USA; RRID:SCR_002798) with the aid of a Biostatistician, using 2-tailed Mann–Whitney *t* tests (Paired, Unpaired) or Tukey’s multiple comparison test using 1-way or 2-way ANOVA analyses, in accordance with the type of data. Treatment group was used as a factor in 1-way ANOVA using GraphPad Prism 10 software (RRID:SCR_002798). The 2 factors used for 2-way ANOVA were treatment groups and cell type, where no interaction was assumed between them using GraphPad Prism 10 software (RRID:SCR_002798). These analyses were done assuming normal distribution of data. Power analysis to determine the number of mice used in this study was based on pilot studies that were conducted prior to the main experiment, ensuring appropriate sample size is used for reliable and statistically significant results. All survival analyses were done using Log-rank (Mantel-Cox) test (using GraphPad Prism 10 software, RRID:SCR_002798) between groups comparing the type of mice and type of treatment. The surviving mice at endpoint were censored. *P* values under 0.05 were taken as significant values. Exact *P* values are mentioned as appropriate.

### Study approval.

All animal studies were conducted per protocols approved by the Institutional animal care and use committee (IACUC) of the Roswell Park and Moffitt Comprehensive Cancer Centers. Patient samples were collected following informed consent under an IRB-approved protocol I-57217 and used in accordance with the Declaration of Helsinki, International Ethical Guidelines for Biomedical Research Involving Human Subjects (CIOMS), Belmont Report, and U.S. Common Rule.

### Data and materials availability.

Values for all data shown in graphs are reported in Supplemental Data Values file. Single cell sequencing data is available on Gene expression omnibus (GSE308219) and Dryad using the following link: https://datadryad.org/dataset/doi:10.5061/dryad.69p8cz9f3

## Author contributions

ECG, JW: Consulting statisticians. PG: Writing – original draft, review and editing, Experiments – conceptualization, design and execution, Data – curation, visualization and analysis. YZ, NB, JCB, CSF: Experiment – execution, Writing – review and editing. CS, AP, SM, DM, MI: Experiment – execution. MJT, SH: Patient sample collection and data procurement, Writing – review and editing. ECG, JW: Single-cell data analysis, Writing – review and editing. SBL, HK: Writing – review and editing. MLS: Writing – review and editing. Experiments – conceptualization. MDJ: Conceptualization, Writing – review and editing. MLD: Supervision, Funding acquisition, Conceptualization, Writing – original draft, review and editing.

## Funding support

This work is the result of NIH funding, in whole or in part, and is subject to the NIH Public Access Policy. Through acceptance of this federal funding, the NIH has been given a right to make the work publicly available in PubMed Central.

- The Mark Foundation for Cancer Research (MD).
- The Rustum Family Endowment for Translational Scientific Achievement (MD).
- Research project RO1 grant NIH-5RO1AI155786-04 (MD).

## Supplementary Material

Supplemental data

Supporting data values

## Version 1 10/30/2025

In-Press Preview

## Version 2 01/02/2026

Electronic publication
