# Supplementary material for: IFN-γ–driven skewing towards Th1 over Th17 differentiation underlies CRS and neutropenia in CAR-T therapy
Source: J Clin Invest. 2025 Oct 30;136(1):e194631. doi: 10.1172/JCI194631 (PMC12721892; doi:10.1172/JCI194631)
Supplement: Supplemental data [file jci-136-194631-s054.pdf]

Supplementary Figures

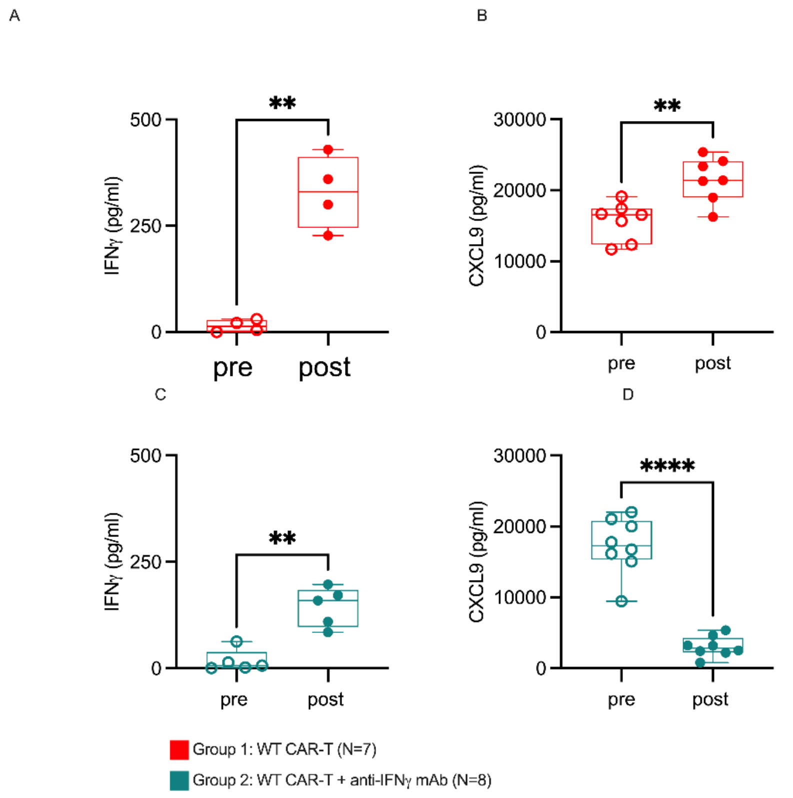

Figure S1: (**A-D**). Comparison of IFNγ (**A, C**) and CXCL9 (**B, D**) between tumor bearing (Eμ-ALL) CAR-T treated IL2Rα KO mice with or without anti-IFNγ mAb. Pre and post cytokine values from (**A-D**) are paired values taken from IL2Rα KO mice that had samples for both pre and post cytokines at week 1 (N=7 for WT CAR-T Group and N=8 for WT CAR-T + anti-IFNγ mAb group). Data from (**A-D**) are from one experiment.

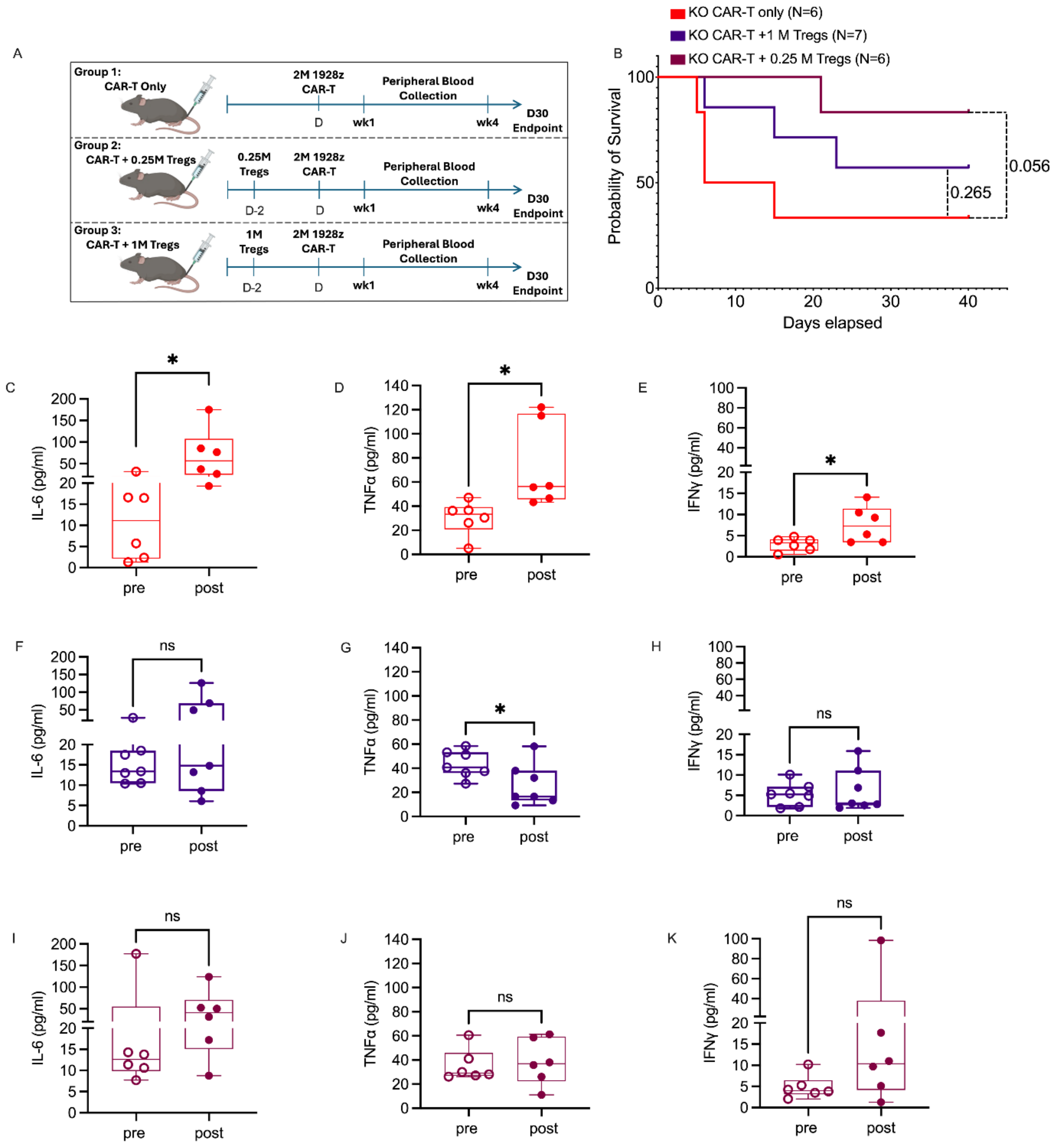

Figure S2: (**A**). Schematic diagram of 2M 1928ζ GFP CAR-T treated KO mice transferred in absence or presence of low (0.25 M) or high dose (1M) Tregs. Data represents a single experiment. (**B**). Kaplan-Meier overall survival curve of CAR-T treated mice in presence or absence of Treg adoptive transfer. (**C-K**). Cytokines IL6 (**C, F, I**), TNFα (**D, G, J**) and IFNγ (**E, H, K**) in CAR-T inoculated mice treated with or without Tregs comparing pre CAR-T vs week 4 levels. Pre and post cytokines are paired values taken from each mouse alive at week 4 or endpoint per mouse. Error bars represent SEM. P values *P < .05, **P < .01, and ***P < .001 were considered significant. P values for cytokine bar plots (**C-K**) were generated using paired t test. P values for Kaplan-Meier survival curve B was generated using Log-rank (Mantel-Cox) test.

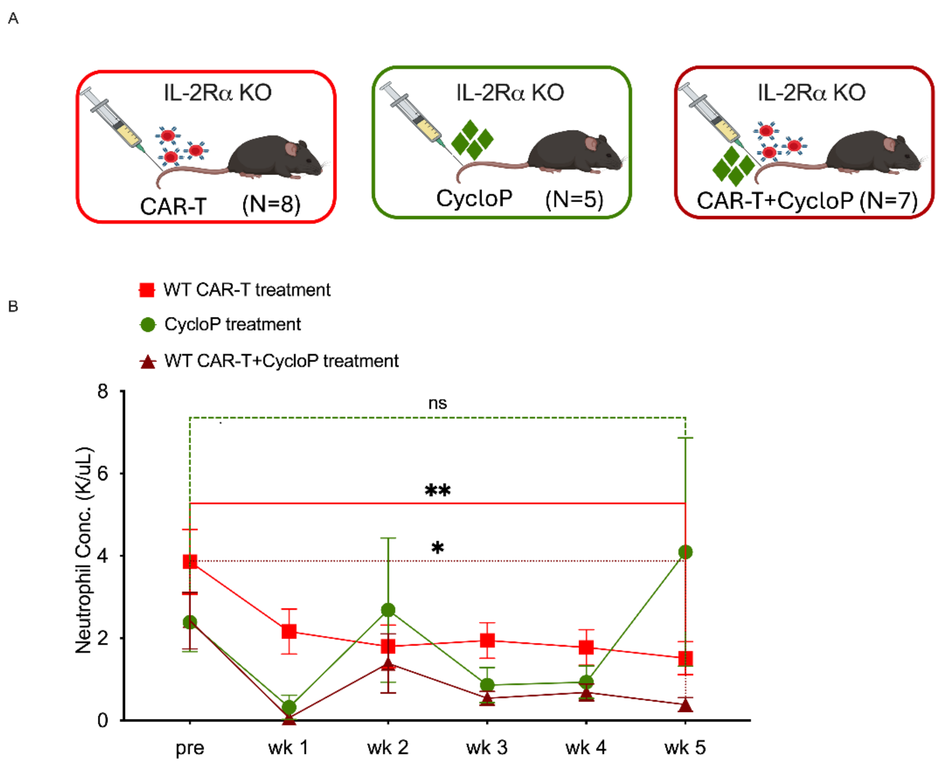

Figure S3: (**A**). Schematic showing KO mice treated with either 2 M 1928ζ cherry CAR-T or 200 mg/kg cyclophosphamide (CycloP) alone or in combination. Differences in recovery rate of Neutrophils were assessed under these 3 different conditions in KO mice. KO mice infused with CAR-T alone are from one of the pooled experiments from Figure 3A that included the two study arms of cyclophosphamide and CAR-T + cyclophosphamide. (**B**). Comparison of neutrophil concentration in the respective groups. Data is pooled from 2 independently performed experiments. Error bars represent SEM. P values *P < .05, **P < .01, and ***P < .001 were considered significant. P values for line plot B was generated using paired t test.

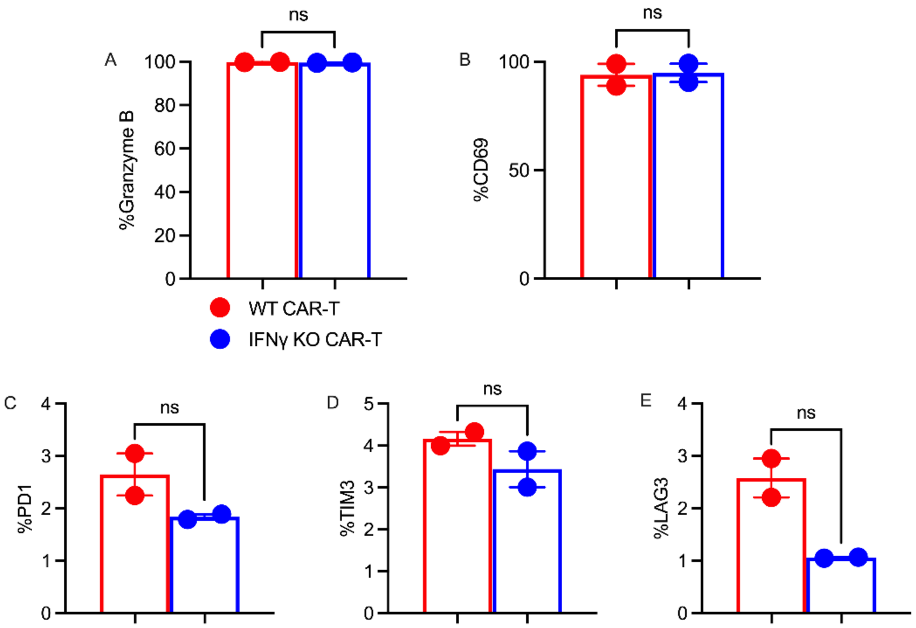

Figure S4: Day 5 transduced 1928ζ GFP CAR-T or IFNγ-/- CAR-T cells were debeaded and treated with Ionomycin for 1 hour, followed by Brefeldin A for 4 hours. Samples were stained intracellularly for activation markers Granzyme B (**A**), CD69 (**B**) as well as exhaustion markers such as PD1 (**C**), TIM3 (**D**) and LAG3 (**E**). Data are from one experiment (N=2). Error bars represent SEM. P values $*P < .05$, $**P < .01$, and $***P < .001$ were considered significant. P values for bar plots A-E were generated using unpaired t test.

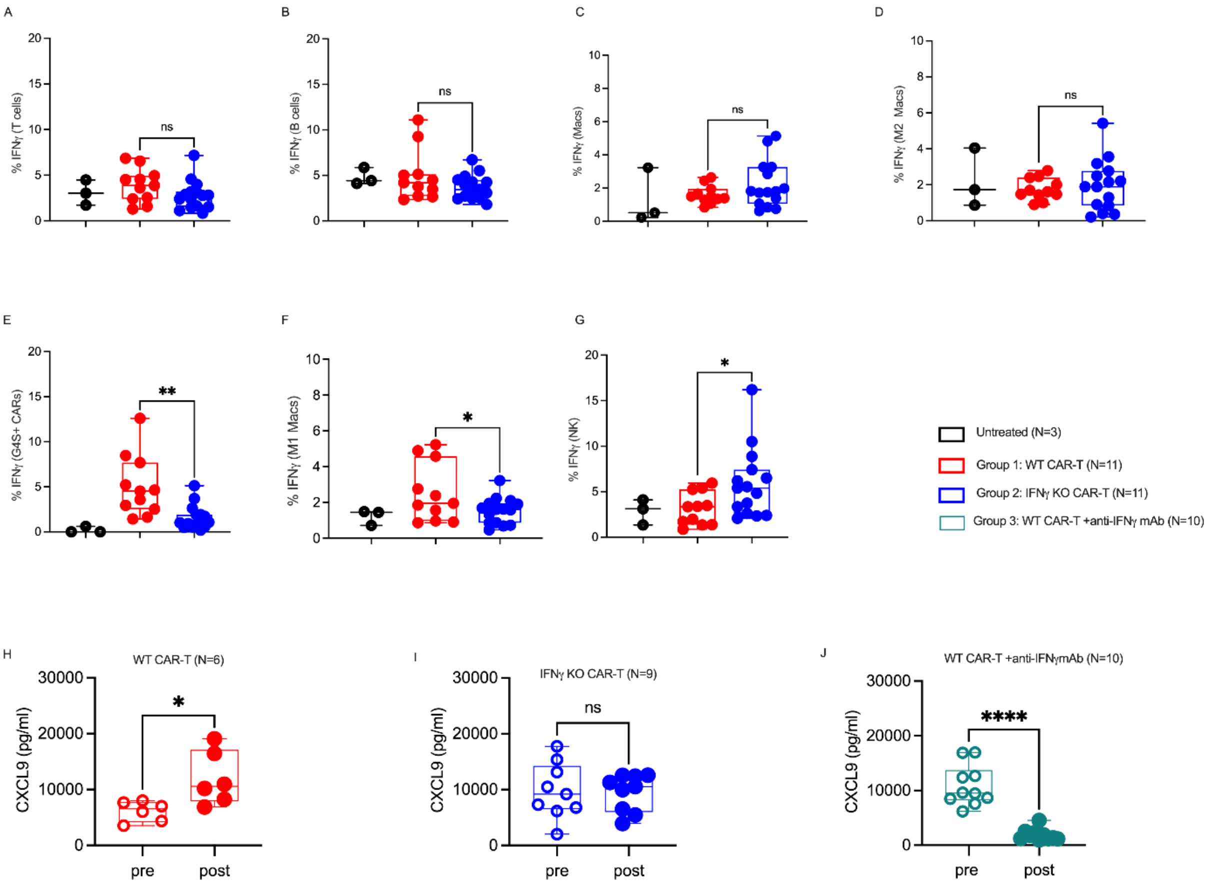

Figure S5: (**A-G**). To determine cellular sources and functionality of IFNγ in IFNγ KO CAR-T treated mice, bone marrow from WT CAR-T (Group 1, N=11) or IFNγ KO CAR-T (Group 2, N=14) treated mice were collected at week 4 and treated with Brefeldin A for 5 hours. Tissues were stained using flow cytometry to detect intracellular IFNγ expression in B, T, NK cells and macrophages. (**H-J**)To determine IFNγ activity CXCL9 concentration was measured in serum collected from peripheral blood of non-tumor bearing IL-2Rα KO mice treated with 2M WT CAR-T (**G**) (Group 1, N=6), IFNγ KO CAR-T (**H**) (Group 2, N=9) and WT CAR-T +anti-IFNγ mAb (**I**) (N=10) at week 4. Data are from one independent experiment. Error bars represent SEM. P values *P < .05, **P < .01, and ***P < .001 were considered significant. P values for (**A-G**) were generated using unpaired t test. P values for (**H-J**) were generated using paired t test.

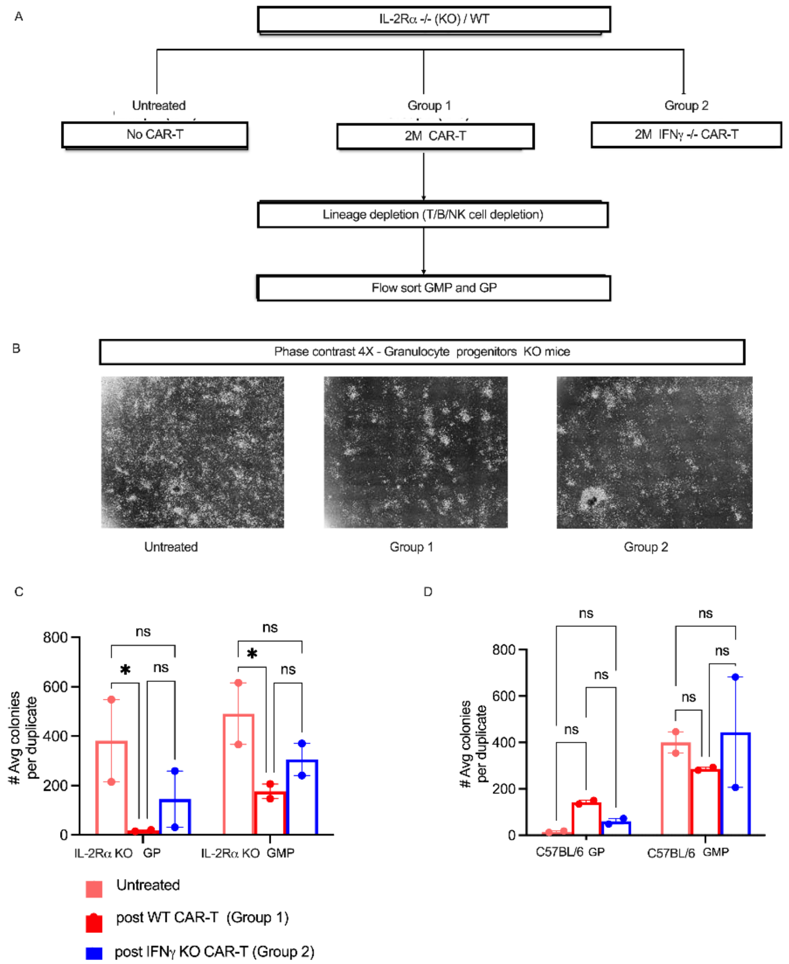

Figure S6: (**A**). Diagrammatic representation of Granulocyte-monocyte-progenitor (GMP) and Granulocyte progenitor (GP) cell isolation for Methocult colony formation. Week 4 post 1928ζ GFP CAR-T (Group 1) or post IFNγ-/- CAR-T (Group 2) treated GMP and GP cells were enriched by Fluorescence-activated cell sorting (FACS) using BMMCs isolated from 4 KO and 4 WT mice per Group. Four untreated mice were used as control. BMMCs were depleted for lineage cells (T/B/NK cells) using kit-based lineage depletion. Lineage negative cell suspensions were FACS sorted to yield ckit+Sca1-FcγR+CD34+Ly6C-Flt3-CD115low GMP cells or ckit+Sca1-CD16/32(FcγR)+CD34+Ly6C+Flt3-CD115low GP cells as described (Methods). Data is represented from a single experiment (KO N=4 per Group, WT N=4 per Group). Sorted GMP cells were pooled from 2 of the 4 mice per group. The cells were cultured ex-vivo on Methocult media for 7 days. The same procedure was followed for GP cells. (**B**). Representative images of a single plate per group displaying the GP colonies formed on Day 7 acquired using phase contrast microscope at 4X magnification. The number of GMP or GP colonies (plated separately) formed in each plate per group for KO (**C**) and WT (**D**), were counted on day 7 using an automated hematopoietic colony counter. Results were reported as average number of colonies formed from duplicates per group. Error bars represent SEM. P values $*P < .05$, $**P < .01$, and $***P < .001$ were considered significant. P values for bar plots (**C**) and (**D**) were generated using Tukey's multiple comparison test (using Two way ANOVA).

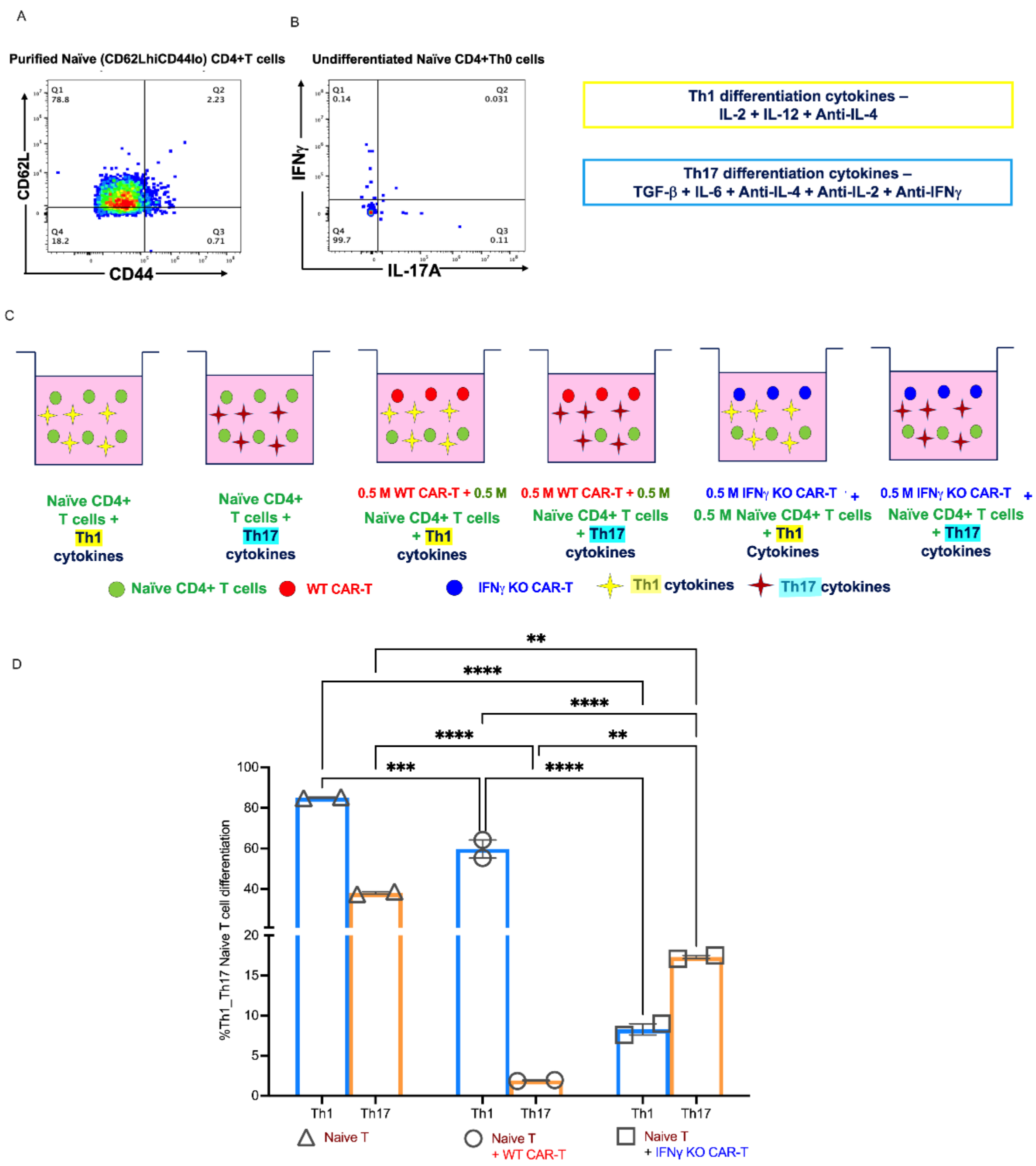

Figure S7: (**A**). Naïve CD4+CD62LhiCD44lo cells were isolated (kit-based purification) and (**B**). evaluated for Th1 cytokine IFNγ (gated as Live+ CD4+IFNγ+) and Th17 cytokine IL-17A (gated as Live+ CD4+ IL-17A+) expression. (**C**). Purified naïve CD4+ T cells were cultured in 24 well plates and differentiated for 4 days into Th1 cells (10ng/mL IL-12, 200IU/mL IL-2 and 1 µg/mL anti-IL4) or Th17 cells (40ng/mL IL-6 , 3ng/mL TGFβ, 1 µg/mL anti-IL4, 1 µg/mL anti-IFNγ and 1 µg/mL anti-IL-2). The differentiating cells were co-cultured alone or in combination with 0.5M 1928ζ GFP CAR-T or IFNγ-/- CAR-T cells. Data is represented from a single experiment (N=2 per condition). (**D**). Bar plots represent the percentage of naïve T cells that differentiated into Th1 (Blue bar plot gated as Live+ CD4+IFNγ+) or Th17 (Yellow bar plot gated as Live+ CD4+ IL-17A+) cells in presence or absence of CAR-T or IFNγ-/- CAR- T cells. Error bars represent SEM. P values *P < .05, **P < .01, and ***P < .001 were considered significant. p values for bar plot (**D**) was generated using Tukey’s multiple comparison test (using Two way ANOVA).

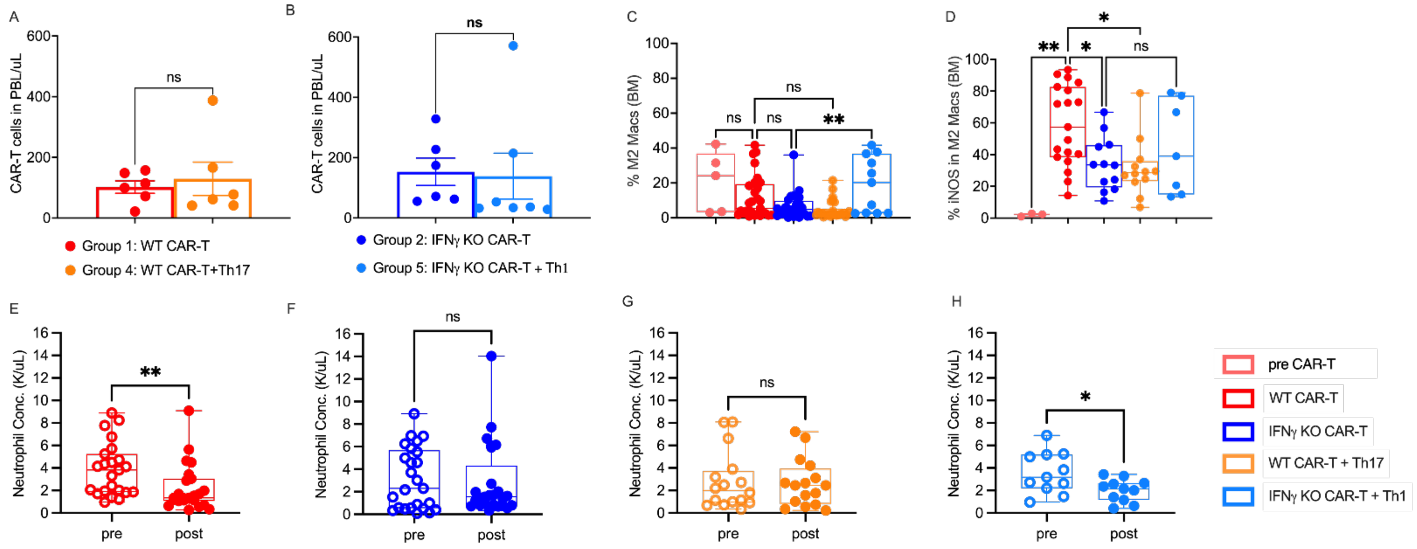

Figure S8: (**A-B**). Effect of Th17 (N=6) and Th1(N=8) on expansion of CAR-T (N=6) and IFNγ KO CAR-T (N=6) was assessed in peripheral blood (CD3+G4S+) of treated mice at week 1 from Figure 5. (**C**). Arginase1+ M2-like Macrophages (gated as Live+ CD45+CD11b+F4/80+CD11c-CD206+Arginase1+ BMMCs), represented as a percent (%) of CD45+ cells were compared in mice alive at week 4 from Group 1 (N=29), Group 2 (N=28), Group 4 (N=26), and Group 5 (N=10) from Figure 5. Data are pooled from 3 independent experiments. (**D**). iNOS+ cells in M2-like macrophages (gated as Live+ CD45+CD11b+F4/80+CD11c-CD206+Arginase1+ iNOS+ BMMCs), represented as a percent (%) of Arginase+ cells were compared in a subset of mice from Groups 1(N=19), 2 (N=12), 4 (N=12), and 5 (N=7) from Figure 5. Data are pooled from 2 independent experiments. (**E– H**) Comparison of circulating neutrophil levels from tumor bearing KO mice in Figure 5 treated with CAR-T cells with or without adoptively transferred Th17 (**E, G**) or IFNγ KO CAR-T cells with or without Th1 (**F, H**) by CBC profiling. Pre and post neutrophil levels are paired values taken from a subset of mice alive at week 4 (Group 1 N=23, Group 2 N=25, Group 4 N= 16 and Group 5 N=11). Error bars represent SEM. P values *P < .05, **P < .01, and ***P < .001 were considered significant. P values for (**A, B**) were generated using unpaired t test. P values for bar plots for M2-like macrophages in (**C, D**) were generated using Tukey's multiple comparison test (using One way ANOVA). P values for neutrophil concentration plots (**E-H**) were generated using paired t test. Data for plots (**A, B**) are from one experiment and (**C-E**) are pooled from 3 independently performed experiments.

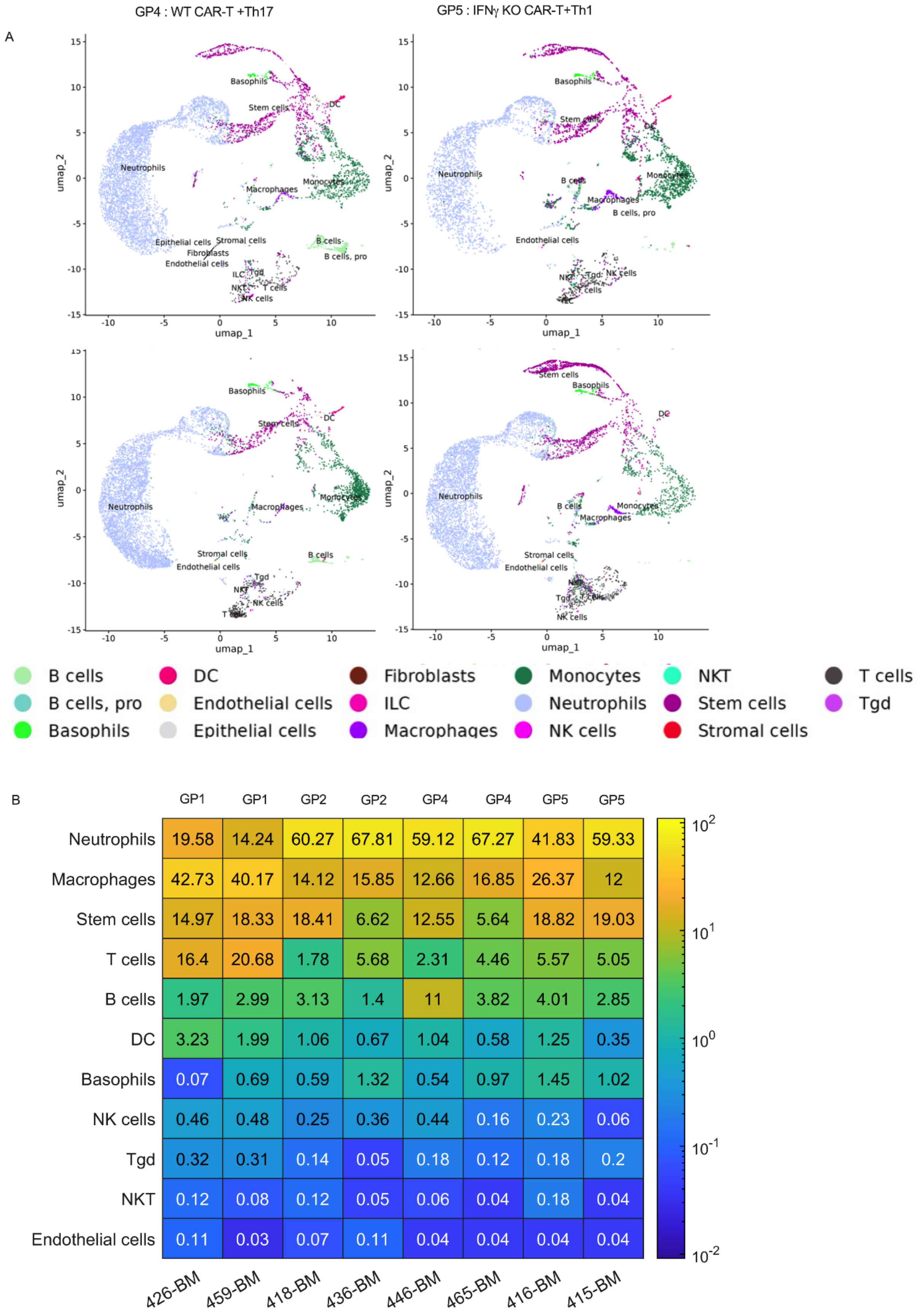

Figure S9: (**A**). UMAPs of individual mice per Group (GP) 4 (CAR-T + Th17) and GP 5 (IFNγ KO CAR-T + Th1) showcasing all cells present in their bone marrow. (**B**). Heat maps represent the frequency of various immune cells as well as stem and endothelial cells per sample in GP 1, 2, 4, and 5 described in Figure 6.

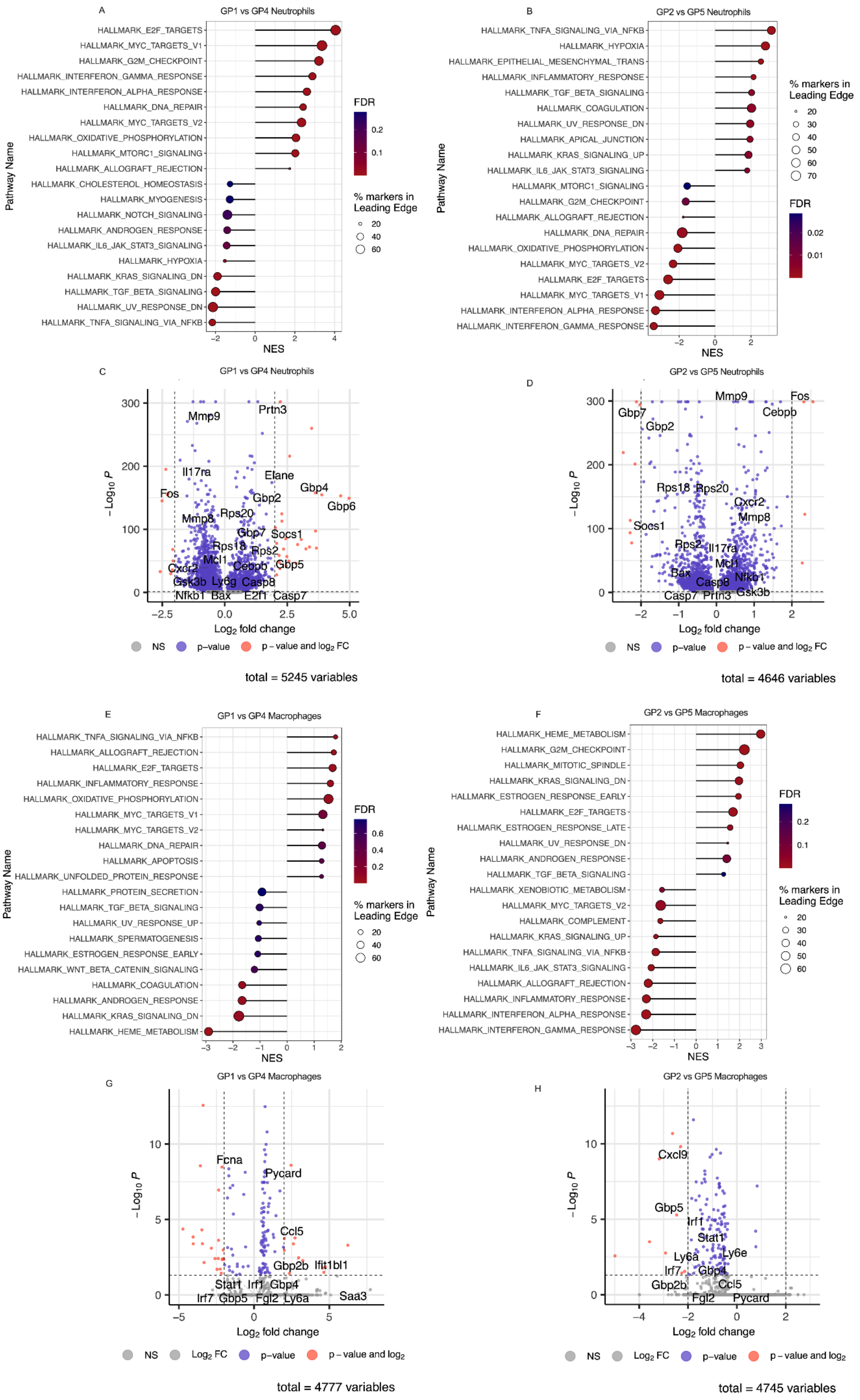
A
GP1 vs GP4 Neutrophils
HALLMARK_E2F_TARGETS
HALLMARK_MYC_TARGETS_V1
HALLMARK_G2M_CHECKPOINT
HALLMARK_INTERFERON_GAMMA_RESPONSE
HALLMARK_INTERFERON_ALPHA_RESPONSE
HALLMARK_DNA_REPAIR
HALLMARK_MYC_TARGETS_V2
HALLMARK_OXIDATIVE_PHOSPHORYLATION
HALLMARK_MTORC1_SIGNALING
HALLMARK_ALLOGRAFT_REJECTION
HALLMARK_CHOLESTEROL_HOMEOSTASIS
HALLMARK_MYOGENESIS
HALLMARK_NOTCH_SIGNALING
HALLMARK_ANDROGEN_RESPONSE
HALLMARK_IL6_JAK_STAT3_SIGNALING
HALLMARK_HYPOXIA
HALLMARK_KRAS_SIGNALING_DN
HALLMARK_TGF_BETA_SIGNALING
HALLMARK_UV_RESPONSE_DN
HALLMARK_TNFA_SIGNALING_VIA_NFKB
Pathway Name
NES
FDR
% markers in Leading Edge
B
GP2 vs GP5 Neutrophils
HALLMARK_TNFA_SIGNALING_VIA_NFKB
HALLMARK_HYPOXIA
HALLMARK_EPITHELIAL_MESENCHYMAL_TRANS
HALLMARK_INFLAMMATORY_RESPONSE
HALLMARK_TGF_BETA_SIGNALING
HALLMARK_COAGULATION
HALLMARK_UV_RESPONSE_DN
HALLMARK_APICAL_JUNCTION
HALLMARK_KRAS_SIGNALING_UP
HALLMARK_IL6_JAK_STAT3_SIGNALING
HALLMARK_MTORC1_SIGNALING
HALLMARK_G2M_CHECKPOINT
HALLMARK_ALLOGRAFT_REJECTION
HALLMARK_DNA_REPAIR
HALLMARK_OXIDATIVE_PHOSPHORYLATION
HALLMARK_MYC_TARGETS_V2
HALLMARK_E2F_TARGETS
HALLMARK_MYC_TARGETS_V1
HALLMARK_INTERFERON_ALPHA_RESPONSE
HALLMARK_INTERFERON_GAMMA_RESPONSE
Pathway Name
NES
% markers in Leading Edge
FDR
C
GP1 vs GP4 Neutrophils
Mmp9
Prtn3
Il17ra
Elane
Fos
Gbp4
Gbp2
Gbp6
Mmp8
Rps20
Gbp7
Socs1
Rps18
Rps2
Mcl1
Cebpb
Gbp5
Cxcr2
Gsk3b
Ly6g
Casp8
Nfkb1
Bax
E2f1
Casp7
$-Log_{10}$ P
Log_2 fold change
NS
p-value
p – value and log_2 FC
total = 5245 variables
D
GP2 vs GP5 Neutrophils
Mmp9
Fos
Gbp7
Cebpb
Gbp2
Rps18
Rps20
Cxcr2
Mmp8
Socs1
Rps2
Il17ra
Mcl1
Bax
Nfkb1
Casp8
Casp7
Prtn3
Gsk3b
$-Log_{10}$ P
Log_2 fold change
NS
p-value
p – value and log_2 FC
total = 4646 variables
E
GP1 vs GP4 Macrophages
HALLMARK_TNFA_SIGNALING_VIA_NFKB
HALLMARK_ALLOGRAFT_REJECTION
HALLMARK_E2F_TARGETS
HALLMARK_INFLAMMATORY_RESPONSE
HALLMARK_OXIDATIVE_PHOSPHORYLATION
HALLMARK_MYC_TARGETS_V1
HALLMARK_MYC_TARGETS_V2
HALLMARK_DNA_REPAIR
HALLMARK_APOPTOSIS
HALLMARK_UNFOLDED_PROTEIN_RESPONSE
HALLMARK_PROTEIN_SECRETION
HALLMARK_TGF_BETA_SIGNALING
HALLMARK_UV_RESPONSE_UP
HALLMARK_SPERMATOGENESIS
HALLMARK_ESTROGEN_RESPONSE_EARLY
HALLMARK_WNT_BETA_CATENIN_SIGNALING
HALLMARK_COAGULATION
HALLMARK_ANDROGEN_RESPONSE
HALLMARK_KRAS_SIGNALING_DN
HALLMARK_HEME_METABOLISM
Pathway Name
NES
FDR
% markers in Leading Edge
F
GP2 vs GP5 Macrophages
HALLMARK_HEME_METABOLISM
HALLMARK_G2M_CHECKPOINT
HALLMARK_MITOTIC_SPINDLE
HALLMARK_KRAS_SIGNALING_DN
HALLMARK_ESTROGEN_RESPONSE_EARLY
HALLMARK_E2F_TARGETS
HALLMARK_ESTROGEN_RESPONSE_LATE
HALLMARK_UV_RESPONSE_DN
HALLMARK_ANDROGEN_RESPONSE
HALLMARK_TGF_BETA_SIGNALING
HALLMARK_XENOBIOTIC_METABOLISM
HALLMARK_MYC_TARGETS_V2
HALLMARK_COMPLEMENT
HALLMARK_KRAS_SIGNALING_UP
HALLMARK_TNFA_SIGNALING_VIA_NFKB
HALLMARK_IL6_JAK_STAT3_SIGNALING
HALLMARK_ALLOGRAFT_REJECTION
HALLMARK_INFLAMMATORY_RESPONSE
HALLMARK_INTERFERON_ALPHA_RESPONSE
HALLMARK_INTERFERON_GAMMA_RESPONSE
Pathway Name
NES
FDR
% markers in Leading Edge
G
GP1 vs GP4 Macrophages
Fcna
Pycard
Ccl5
Gbp2b
Ifit1bl1
Stat1
Irf1
Gbp4
Irf7
Gbp5
Fgl2
Ly6a
Saa3
$-Log_{10}$ P
Log_2 fold change
NS
Log_2 FC
p-value
p – value and log_2
total = 4777 variables
H
GP2 vs GP5 Macrophages
Cxcl9
Gbp5
Irf1
Stat1
Ly6a
Ly6e
Irf7
Gbp4
Gbp2b
Ccl5
Fgl2
Pycard
$-Log_{10}$ P
Log_2 fold change
NS
Log_2 FC
p-value
p – value and log_2
total = 4745 variables

Figure S10: (**A-D**). Comparison of gene set enrichment analysis showing hallmark pathways and respective volcano plots showing differentially expressed genes in Neutrophils from GP 1 vs GP 4 (**A, C**) and GP 2 vs GP 5 (**B, D**). (**E-H**). Comparison of gene set enrichment analysis showing hallmark pathways and respective volcano plots showing differentially expressed genes in Macrophages from GP 1 vs GP 4 (**E, G**) and GP 2 vs GP 5 (**F, H**).

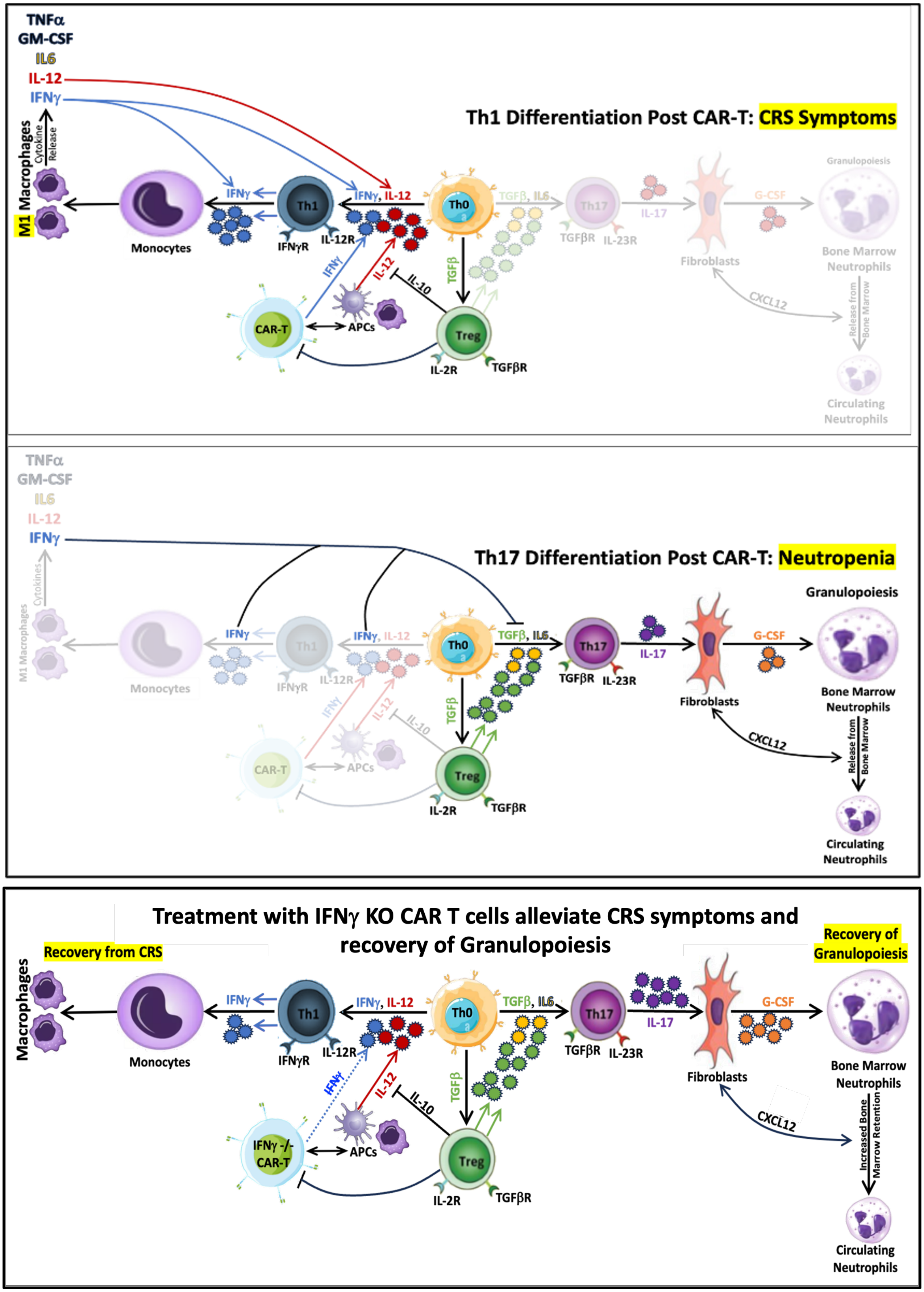

Figure S11: Putative mechanism showing how IFNγ regulates Th1 (**A**) and Th17 axis (**B**) leading to CRS-neutropenia symptoms that are recovered with IFNγ KO CAR-T treatment (**C**).

Supplementary Table S1: Baseline demographic and clinical characteristics per patient.

NHL:non-Hodgkin lymphoma; MM:Multiple myeloma; DLBCL GCB: Germinal Center B-Cell-Like Diffuse Large B-Cell Lymphoma; DLBC ABC: Activated B-cell-like diffuse large B-cell lymphoma; PCL: Plasma cell leukemia. CR:complete response; PR:partial response; PROG:progression; VGPR:very good partial response; NE: Not evaluable.

Patient #	Disease & Diagnosis	Age (years)	Gender	CAR-T product	Best response
1	NHL, Mantle	48 Years	Male	Brexucabtagene autoleucel	CR
2	NHL, DLBC ABC	68 Years	Male	Axicabtagene ciloleucel	CR
3	NHL, DLBC GCB	42 Years	Male	Lisocabtagene maraleucel	NE
4	NHL, DLBC ABC	58 Years	Male	Lisocabtagene maraleucel	CR
5	MM, None, Lambda, IIIA	40 Years	Male	Idecabtagene vicleucel	CR
6	MM, kappa FLC	62 Years	Male	Idecabtagene vicleucel	VGPR
7	NHL, Mantle	61 Years	Male	Brexucabtagene autoleucel	CR
8	NHL, DLBC ABC	72 Years	Male	Lisocabtagene maraleucel	CR
9	NHL, DLBC GCB	59 Years	Male	Axicabtagene ciloleucel	CR
10	NHL, Mantle	70 Years	Male	Brexucabtagene autoleucel	CR
11	NHL, Mantle	50 Years	Male	Brexucabtagene autoleucel	PROG
12	MM, IgG, Kappa, IIIA	66 Years	Female	Idecabtagene vicleucel	PR
13	MM, IgD, Lambda, IIIA	49 Years	Female	Idecabtagene vicleucel	PROG
14	MM, IgG, Kappa, IIIA	68 Years	Male	Ciltacabtagene autoleucel	VGPR
15	MM, IgG, Lambda, IIA	42 Years	Female	Idecabtagene vicleucel	PROG
16	MM, IgG, Kappa, IIIA	74 Years	Male	Idecabtagene vicleucel	PROG
17	NHL, DLBC GCB	60 Years	Male	Tisagenlecleucel	PROG
18	NHL, DLBC GCB	36 Years	Female	Tisagenlecleucel	PROG
19	NHL, Mantle	56 Years	Male	Brexucabtagene autoleucel	CR
20	MM, IgG, Kappa, IIIA	60 Years	Female	Idecabtagene vicleucel	PROG
21	NHL, BcellNOS	73 Years	Male	Lisocabtagene maraleucel	PR
22	MM, Non-sec, IIIA	67 Years	Female	Idecabtagene vicleucel	PROG
23	NHL, DLBC ABC	69 Years	Male	Axicabtagene ciloleucel	CR
24	NHL, DLBC NOS	49 Years	Male	Tisagenlecleucel	PROG
25	NHL, DLBC ABC	52 Years	Female	Tisagenlecleucel	PROG
26	NHL, DLBC ABC	74 Years	Male	Tisagenlecleucel	CR
27	NHL, DLBC GCB	48 Years	Male	Tisagenlecleucel	PROG
28	NHL, DLBC ABC	57	Male	axicabtagene ciloleucel	CR

29	PCL, NOS	56	Female	ciltacabtagene autoleucel	CR
30	MM, IgG lambda	71	Male	ciltacabtagene autoleucel	PR
31	NHL, DLBC GCB	78	Female	lisocabtagene maraleucel	CCR
32	MM, IgG kappa	66	Male	ciltacabtagene autoleucel	PR
33	MM, IgG kappa	56	Female	ciltacabtagene autoleucel	PR
34	NHL,FollGrIIIa	65	Male	axicabtagene ciloleucel	PROG
35	NHL, DLBC ABC	69	Male	axicabtagene ciloleucel	CR
36	NHL, FollGrIIIa	58	Male	axicabtagene ciloleucel	CR
37	MM, IgG lambda	55	Male	ciltacabtagene autoleucel	PR
38	NHL, DLBC ABC	65	Female	lisocabtagene maraleucel	CR
39	MM, IgG kappa	57	Female	ciltacabtagene autoleucel	SD
40	MM, IgG lambda	67	Male	ciltacabtagene autoleucel	CR
41	NHL, Mantle	62	Male	lisocabtagene maraleucel	PROG
42	MM, IgA kappa	71	Male	ciltacabtagene autoleucel	PR
43	NHL, DLBC ABC	71	Male	lisocabtagene maraleucel	CR

Supplementary Table S2: Patient classification based on risk of developing CRS and neutropenia

Patient #	Severity-based neutropenia (*69*)	Recovery-based neutropenia (*68)*	CRS	Max Grade CRS	Risk based group classification
1	Prolonged neutropenia	Intermittent recovery	yes	1	Low-grade-CRS-Neutropenia
2	Prolonged neutropenia	Intermittent recovery	no	0	No cooccurence
3	None	Quick recovery	yes	1	No cooccurence
4	Profound neutropenia	Quick recovery	no	0	No cooccurence
5	Prolonged neutropenia	Intermittent recovery	yes	1	Low-grade-CRS-Neutropenia
6	Protracted + Prolonged	Intermittent recovery	yes	1	Low-grade-CRS-Neutropenia
7	None	Quick recovery	yes	2	No cooccurence
8	None	Quick recovery	no	0	No cooccurence
9	Prolonged neutropenia	Intermittent recovery	no	0	No cooccurence
10	Prolonged neutropenia	Intermittent recovery	yes	2	High-grade-CRS-Neutropenia
11	None	Quick recovery	no	0	No cooccurence
12	Profound + Protracted + Prolonged	Aplastic neutropenia	yes	2	High-grade-CRS-Neutropenia
13	None	Quick recovery	no	0	No cooccurence
14	Prolonged + profound	Intermittent recovery	yes	3	High-grade-CRS-Neutropenia
15	Prolonged neutropenia	Not available	no	0	No cooccurence
16	Protracted + Prolonged	Intermittent recovery	no	0	No cooccurence
17	None	Quick recovery	no	0	No cooccurence
18	Prolonged neutropenia	Intermittent recovery	yes	2	High-grade-CRS-Neutropenia
19	Protracted neutropenia	Intermittent recovery	yes	2	Low-grade-CRS-Neutropenia
20	Protracted + Profound	Quick recovery	yes	2	No cooccurence
21	Prolonged neutropenia	Intermittent recovery	no	0	No cooccurence
22	Profound + Protracted + Prolonged	Aplastic neutropenia	yes	1	Low-grade-CRS-Neutropenia
23	Prolonged neutropenia	Intermittent recovery	yes	3	High-grade-CRS-Neutropenia
24	None	Quick recovery	no	0	No cooccurence
25	None	Quick recovery	no	0	No cooccurence
26	Protracted neutropenia	Quick recovery	no	0	No cooccurence
27	Prolonged neutropenia	Intermittent recovery	yes	1	Low-grade-CRS-Neutropenia
28	None	Quick recovery	no	0	No cooccurence
29	None	Quick recovery	yes	2	No cooccurence
30	Prolonged	Intermediate+Aplastic recovery	yes	1	Low-grade-CRS-Neutropenia
31	Prolonged	Aplastic recovery	no	0	No cooccurence
32	Protracted	Intermediate recovery	yes	2	High-grade-CRS-Neutropenia
33	Protracted	Quick recovery	yes	1	No cooccurence
34	Protracted	Intermediate recovery	yes	2	High-grade-CRS-Neutropenia

35	None	Quick recovery	yes	2	No cooccurence
36	None	Quick recovery	no	0	No cooccurence
37	Prolonged	Aplastic recovery	no	0	No cooccurence
38	None	Quick recovery	yes	2	No cooccurence
39	Protracted	Aplastic recovery	yes	2	High-grade-CRS-Neutropenia
40	None	Quick recovery	no	0	No cooccurence
41	Protracted	Intermediate recovery	no	0	No cooccurence
42	Protracted	Quick recovery	no	0	No cooccurence
43	None	Quick recovery	no	0	No cooccurence

Supplementary Table S3: Antibody panel for CAR-T expansion in peripheral blood

Marker	Clone	Fluorophore	Source	Catalogue No.
CD3	145-2C11	BUV395	BD Biosciences	565992
CD19	ID3/CD19	BV650	Biolegend	152427
G4S	E702V	APC	Cell Signaling Technology	68718S
Thy1.1	OX-7	PE	Biolegend	202518
GFP		GFP		
Zombie NIR			Biolegend	423105

Supplementary Table S4: Antibody panel for neutrophil maturation

Marker	Clone	Fluorophore	Source	Catalogue No.
CD3	17A2	Percp cy5.5	Biolegend	100218
Nk1.1	PK136	BV605	Biolegend	108753
B220	RA3-6B2	BUV395	BD Biosciences	563793
c-kit	S18020H	PE-cy7	Biolegend	161612
CD11b	M1/70	BUV737	BD Biosciences	612800
CD115	W19330C	PE	Biolegend	165003
Gr-1	RB6-8C5	BV510	Biolegend	108457
Ly6G	1A8	BV785	Biolegend	127645
CXCR4	L276F12	BV711	Biolegend	146517
CXCR2	SA044G4	APC	Biolegend	149312
Zombie NIR			Biolegend	423105

Supplementary Table S5: Antibody panel for bone marrow macrophage characterization

Marker	Clone	Fluorophore	Source	Catalogue No.
CD45	QA17A26	Percp cy5.5	Biolegend	157612
F4/80	BM8	BV421	Biolegend	123132
CD11b	M1/70	BUV737	BD Biosciences	612800
Ly6G	1A8	BV785	Biolegend	127645
Ly6C	HK1.4	BV570	Biolegend	128030
CD115	AFS98	BV711	Biolegend	135515
MHCII	2G9	BV480	BD Biosciences	746669
CD86	GL1	FITC	BD Biosciences	561962
Siglec F	S17007L	PE-Dazzle 594	Biolegend	155530
CD11c	N418	BUV563	BD Biosciences	749040
iNOS	W16030C	PE	Biolegend	696806
CD206	Y17-505	BUV395	BD Biosciences	568817
Arg-1	A1exF5	PE-cy7	e-Bioscience	25-3697-82
Zombie NIR			Biolegend	423105

Supplementary Table S6: Antibody panel for granulocyte monocyte progenitor cell characterization

Marker	Clone	Fluorophore	Source	Catalogue No.
CD3	17A2	Percp cy5.5	Biolegend	100218
B220	RA3-6B2	BUV395	BD Biosciences	563793
Nk1.1	PK136	BV605	Biolegend	108753
c-kit	S18020H	PE-cy7	Biolegend	161612
CD16/32	S17011E	PE-Dazzle 594	Biolegend	156616
CD34	RAM34	FITC	BD Biosciences	560238
Sca-1	W18174A	APC	Biolegend	160904
Ly6C	HK1.4	BV570	Biolegend	128030
Flt3	A2F10	PE	Biolegend	135306
CD115	AFS98	BV711	Biolegend	135515
Zombie NIR			Biolegend	423105

Supplementary Table S7: Antibody panel for characterizing Th1 and Th17

Marker	Clone	Fluorophore	Source	Catalogue No.
CD3	17A2	BV650	Biolegend	100229
CD4	RM4-5	BV785	Biolegend	100552
CD62L	W18021D	Percp cy5.5	Biolegend	161210
CD44	IM7	BV421	Biolegend	103039
IFNγ	XMG1.2	BV711	Biolegend	505836
IL-17A	TC11-18H10.1	APC	Biolegend	506916
Thy1.1	OX-7	PE	Biolegend	202518
GFP		GFP		
Zombie NIR			Biolegend	423105
